# Morphological and cytoskeleton changes in cells after EMT

**DOI:** 10.1038/s41598-023-48279-y

**Published:** 2023-12-13

**Authors:** Assel Nurmagambetova, Vadim Mustyatsa, Aleena Saidova, Ivan Vorobjev

**Affiliations:** 1 School of Sciences and Humanities, Nazarbayev University, Kabanbay Batyr Avenue, 53, 010000 Astana, Kazakhstan; 2 School of Engineering and Digital Sciences, Nazarbayev University, Kabanbay Batyr Avenue, 53, 010000 Astana, Kazakhstan; 3 National Laboratory Astana, Nazarbayev University, Kabanbay Batyr Avenue, 53, 010000 Astana, Kazakhstan

**Keywords:** Biological techniques, Biotechnology, Cancer, Cell biology

## Abstract

Epithelial cells undergoing EMT experience significant alterations at transcriptional and morphological levels. However, changes in the cytoskeleton, especially cytoskeleton dynamics are poorly described. Addressing the question we induced EMT in three cell lines (MCF-7, HaCaT and A-549) and analyzed morphological and cytoskeletal changes there using immunostaining and life cell imaging of cells transfected with microtubule and focal adhesion markers. In all studied cell lines, cell area after EMT increased, MCF-7 and A-549 cells became elongated, while HaCaT cells kept the aspect ratio the same. We next analyzed three components of the cytoskeleton: microtubules, stress fibers and focal adhesions. The following changes were observed after EMT in cultured cells: (i) Organization of microtubules becomes more radial; and the growth rate of microtubule plus ends was accelerated; (ii) Actin stress fibers become co-aligned forming the longitudinal cell axis; and (iii) Focal adhesions had decreased area in all cancer cell lines studied and became more numerous in HaCaT cells. We conclude that among dynamic components of the cytoskeleton, the most significant changes during EMT happen in the regulation of microtubules.

## Introduction

Epithelial-to-mesenchymal transition (EMT) is an important process that occurs during normal development and contributes to pathological processes (e.g. cancer progression and metastases)^1–4^. During EMT epithelial cells lose cell–cell contact, obtain increased motility and invasiveness^5,6^. The gold standard of EMT confirmation includes morphological changes^7,8^ as well as changes in transcriptional profile^9^.

Changes in transcriptional profile are numerous, including downregulation of E-cadherin expression, responsible for cell–cell adhesions, and upregulation of vimentin and fibronectin expression that facilitate increased cell–matrix interactions^10,11^. While the transcriptional aspects of EMT have been well studied and transcription factors like Zeb, Snail and Twist have been identified as key drivers of this process, the description of morphological processes accompanying EMT is much less detailed and mechanisms underlying the changes in cell shape require further research^12^. Morphological changes during the EMT of epithelial cells are often described as a loss of cobblestone-like form with apico-basal polarity and a gain of elongated fusiform shape typical for mesenchymal cells^13^.

The changes in cell shape and morphology always come from the synergistic contribution of the two dynamic components of the cytoskeleton: actin filaments and microtubule network^14^.

The contribution of actin cytoskeleton is relatively well studied. It has been described that rearrangements of F-actin influence cell morphology, migration and invasion characteristics^15,16^. For TGF-β-induced EMT, the remodeling of the actin cytoskeleton requires the activation of the RhoA, which stimulates the assembly of actin filaments into stress fibers and it is also necessary to disrupt E-cadherin at cell–cell adhesions promoting mesenchymal cell morphology^17–19^.

The interaction between actin cytoskeleton and extracellular matrix occurs mainly through focal adhesions. Focal adhesions are involved in the regulation of mesenchymal cell adhesion and migration, and upregulation of focal adhesion kinase (FAK) during EMT is associated with increased cell motility^20,21^. However, the overall description of FA behavior during EMT is lacking.

Microtubules are another highly dynamic component of the cytoskeleton. Inhibition of microtubule dynamics with paclitaxel or modest dosages of nocodazole dramatically impairs cell motility^22^. Even subtle changes in microtubule dynamics can influence cell migration through a variety of microtubule-dependent pathways^23–25^. However, the input of microtubule dynamics in EMT remains largely unknown.

Thus, the detailed analysis of the cytoskeleton's role in EMT requires further investigation.

In the current study, we explored the role of the microtubule network by looking at microtubule dynamics and spatial organization, evaluated the role of actin cytoskeleton by looking at the distribution of actin filaments and analyzed focal adhesion formation and turnover. We show how the dynamics of microtubules and focal adhesions are affected by the induction of EMT in cells of different origins.

## Results

### Morphological changes after induction of EMT

**Figure 1:**
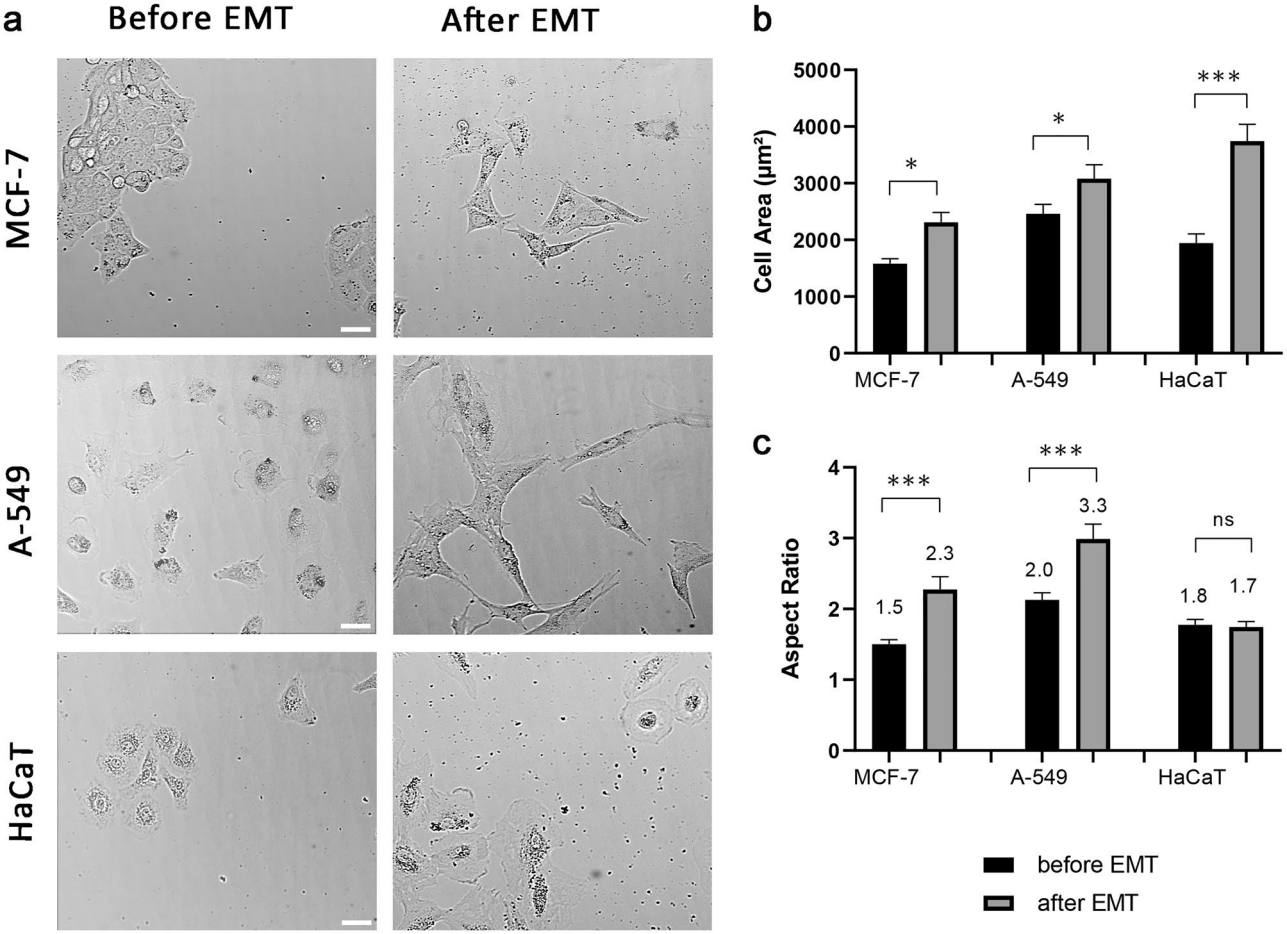
Morphological changes after EMT in three cell lines. (**a**) Brightfield image of morphological changes after EMT. Left picture- MCF-7, A-549 and HaCaT cells before EMT (untreated). Right picture- MCF-7 after EMT (treated) with the addition of doxycycline (10 µg/mL for 48–72 h), A-549 and HaCaT cells after EMT treated with TGF-β (5 ng/mL for 72 h). EMT stimulation caused cell morphology change. Cells became spreader; a few lost cell–cell contact and became elongated. Scale bar 10 µm. (**b**) Differences in cell area in cancer cells before and after EMT. After EMT, cell area increases in all three cell cultures. (**c**) The differences in aspect ratio in different cancer cells before and after EMT. After EMT, MCF-7 and A-549 cells became more elongated. Significance *p < 0.05; ***p < 0.001. The error bars represent mean ± SEM values.

EMT was induced in three different cell lines: breast cancer cells MCF-7, lung cancer cells A-549 and immortalized normal human keratinocytes HaCaT. MCF-7 cells were treated with doxycycline at a final concentration of 10 µg/mL every 24 h for 3 days, as described elsewhere^26^. The efficiency of the launched EMT process in cells was assessed by the expression of Zeb1-GFP fusion protein, which was visualized by GFP fluorescence (Supplementary Fig. S1). EMT in A549 and HaCaT cells was initiated by treatment with TGF-β1 for 3 days, as described elsewhere^27^. After EMT, the following morphological changes were observed in all three cell lines: cells increased in the area: MCF-7 cells by 46%; A549 cells by 25% and HaCaT cells by 93%, and formed more protrusions (Fig. 1a). MCF-7 and A549 cells became elongated (Fig. 1b,c and Supplementary Table S1). Consistent with morphological data, major EMT characteristics for all three cell lines were confirmed by western blot and RT PCR data (Supplementary Fig. S2). Western blotting showed that the expression of the E-cadherin was decreased in A-549 cells after EMT compared with the cells before EMT. Increases in the expression of the mesenchymal marker (N-cadherin) and EMT-related transcription factor (Slug) were also upregulated in transformed A-549 cells. E-cadherin expression in MCF-7 and HaCaT cells remained unchanged after EMT, while the expression of N-cadherin and Slug were slightly upregulated in MCF-7 and HaCaT cells after EMT (cropped blots are presented in Supplementary Fig. S2a; original gels are presented in Supplementary Fig. S3).

Real-time PCR showed that the expression of E-cadherin decreased in A-549 and MCF-7 cells after EMT compared with the cells before EMT. Increases in the expression of the mesenchymal markers (N-cadherin, vimentin) and EMT-related transcription factors (Snail, Twist and Slug) were also observed (Supplementary Fig. S2b,c). Furthermore, increased expression of the EMT-related mesenchymal markers (N-cadherin, vimentin) and EMT-related transcription factors (Snail, Twist and Slug) was observed in HaCaT cells after EMT compared with HaCaT cells before EMT. No significant changes were observed in levels of the epithelial adhesion molecule E-cadherin (Supplementary Fig. S2d).

Since it was recently supposed that not all cells might undergo EMT simultaneously and thus display divergent phenotypes^28^, we calculated the variability of morphological parameters before and after EMT in our cell models. It slightly increased for MCF-7 and A-549 cells and even decreased for HaCaT cells (Supplementary Table S1).

### Changes in microtubule array after EMT

To identify the differences in MT array before and after EMT, we performed immunofluorescent analysis of fixed cells and the analysis of MT growth using live cell imaging of EB3 transfected cells.

**Figure 2:**
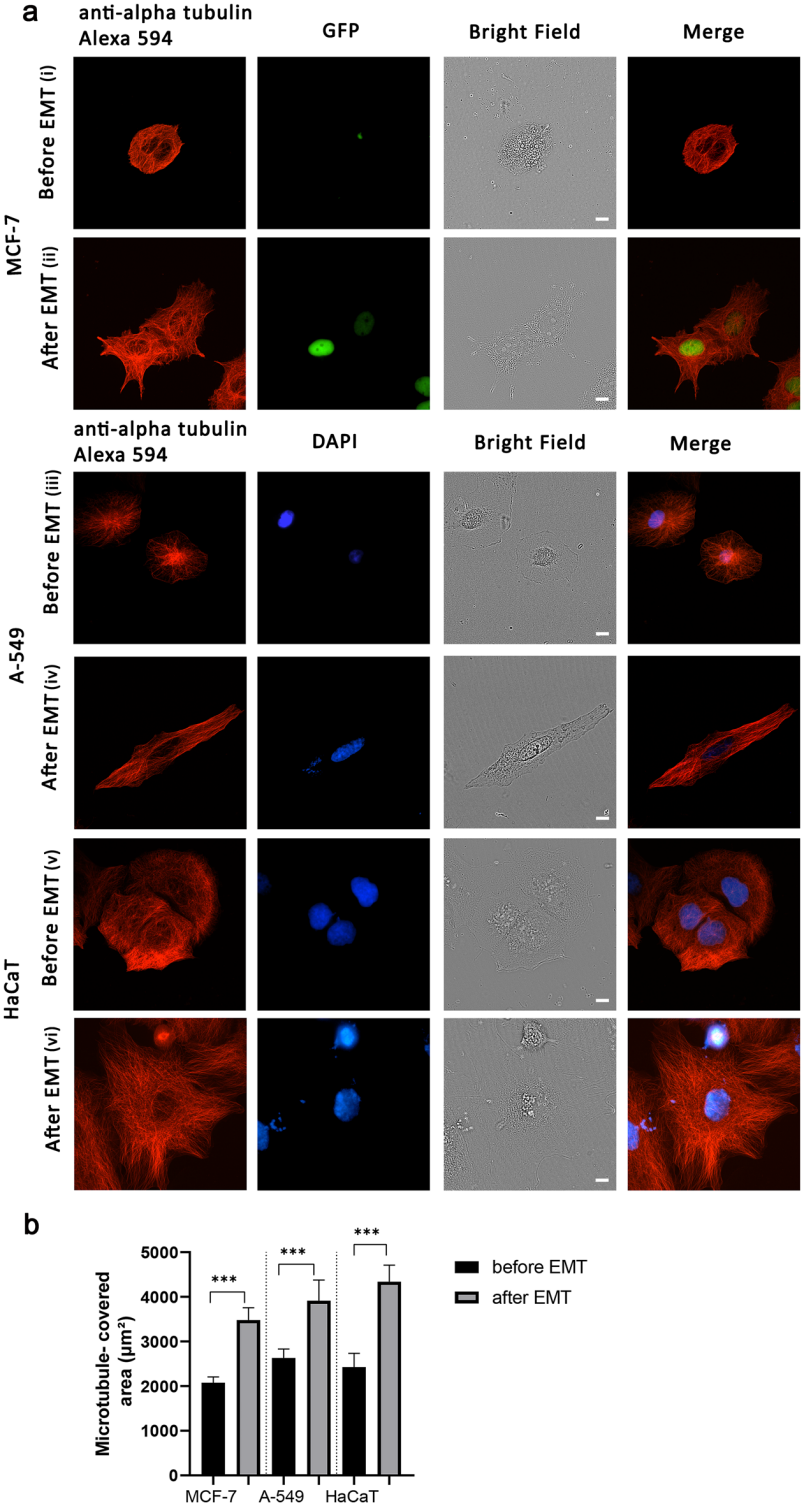
Comparison of microtubule distribution in cells before and after EMT. (**a**) Immunofluorescent confocal microscopy images of MCF-7, A-549 and HaCaT cells before EMT (i, iii, v). MCF-7, A-549 and HaCaT cells after EMT (ii, iv, vi). Cells were stained with Alexa Fluor 594 Anti-alpha Tubulin antibody [EP1332Y]—Microtubule Marker (ab202272) (red) and DAPI (blue). GFP (green) as an indicator of EMT-induced process in MCF-7 cells. Scale bar 10 µm. (**b**) Changes in the microtubule-covered area of cells after EMT, where microtubule organization was analyzed. MCF-7 cells before EMT (N = 42 cells), MCF-7 cells after EMT (N = 47 cells); A-549 cells before EMT (N = 87 cells), A-549 cells after EMT (N = 45 cells); HaCaT cells before EMT (N = 49 cells), HaCaT cells after EMT (N = 45 cells). Significance ***p < 0.001. The error bars represent mean ± SEM values.

The distribution of microtubules at the edge in cells after EMT changed, so that more microtubules were mainly running perpendicular to the cell edge (Fig. 2aii,iv,vi), while before EMT MTs were mainly running along the cell edge (Fig. 2ai,iii,v) (Supplementary 3D reconstruction).

For detailed quantitative evaluation of the distribution of MTs the following criteria were considered: “microtubule-covered area” (Supplementary Fig. S4), “circularity” (Supplementary Fig. S5), “individual MTs at cell edges” (Supplementary Fig. S6) and “nucleus coverage” (Supplementary Fig. S7).

After EMT, the microtubule-covered area increased significantly in all cell cultures analyzed. The microtubule-covered area in MCF-7 cells increased by 67% (Fig. 2b); In A-549 cells, it increased by 49%. In HaCaT cells, it increased by 79%. This is in agreement with that the area of all three cell lines increased after EMT.

Changes in the other parameters related to microtubules were different depending on the cell culture (Supplementary Fig. S8). Individual MTs at the cell edge were mostly identified in MCF-7 and HaCaT cells after EMT, whilst individual MTs in A-549 cells were visible both before and after EMT (Supplementary Fig. S8a). MTs at the cell edge being less intertwined and more perpendicular to the edge likely correspond to a more active extension of protrusion after EMT, with the cytoskeleton being only recently formed there. The circularity parameter after EMT did not change considerably, except for A-549 cells where it decreased by 22% (Supplementary Fig. S8b). Changes in the circularity of the microtubule-covered area were in accord with the overall changes in the cell shape in A549, MCF-7 and HaCaT cells observed after EMT. Nucleus coverage by MTs decreased in A-549 cells after EMT by 50%, but did not change considerably in HaCaT cells (Supplementary Fig. S8c).

**Figure 3:**
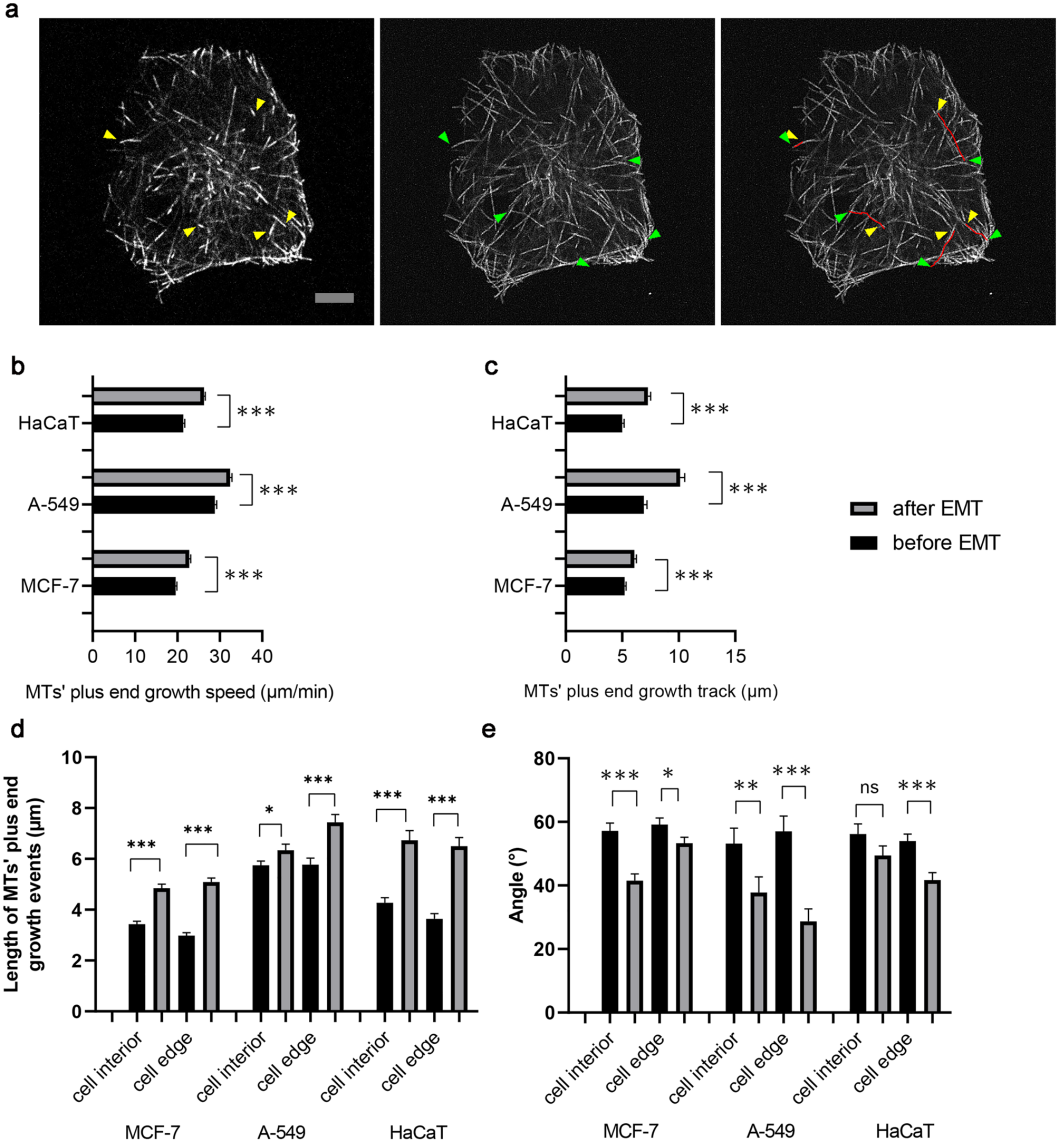
Four characteristics of plus end growing microtubules. Speed of plus end growing microtubules based on tracking of the fluorescently labeled protein EB-3. (**a**) The left image of the figure shows the beginning of a time-lapse film of MTs’ growth. The right image shows Maximum Intensity Projection (MIP) images of 20 s of the time-lapse, which includes 10 frames of the video. The yellow arrows indicate the positions of EB3 comets at the beginning of a time-lapse, while the green arrows indicate their positions after 20 s. Red lines are used to indicate the tracks of the comet's growth. Scale bar 10 µm. (**b**) Differences in MTs’ plus end growth speed before and after EMT. (**c**) Length of MTs’ plus end growth tracks in three different cell lines before and after EMT. (**d**) The differences in MTs’ plus end growth events before and after EMT. (**e**) The differences in an angle of plus end growing MTs’ with respect to cell radius. The number of events analyzed and cell numbers are indicated in Supplementary material Table S3. Significance ***p < 0.001, **p < 0.01; *p < 0.05. The error bars represent mean ± SEM values.

Next, the dynamics of microtubule growth were evaluated by tracking EB-3 comets by analyzing the following parameters, namely plus end growth velocity; length of continuous growth tracks and an angle of plus end growth trajectory with respect to cell radius. Mean values of the first two parameters increased significantly after EMT in all three examined cell lines (Fig. 3b,c).

The velocity of MTs growth in MCF-7 cells after EMT increased by 15%; in A-549 cells by 10% and in HaCaT cells—by 18% (Fig. 3b). The length of MTs’ plus end growth tracks in MCF-7 cells after EMT increased by 15%, in A-549 cells—by 49%, and in HaCaT cells—by 45% (Fig. 3c, Supplementary Table S2).

The next parameter was an angle of plus end growth trajectory with respect to cell radius. After transfection with EB-3-RFP in the majority of cells centrosome area was clearly seen on the maximal intensity projection picture (Supplementary Fig. S9) it was possible to determine cell radius as a direction from the centrosome towards the beginning of the growth track (Supplementary Fig. S9, enlargements).

More detailed analysis of these parameters in the cell interior and near the cell edge show that: (i) density of growing MTs (number of comets per unit cell area) did not change after EMT in all three cultures (Supplementary Table S3); (ii) the persistence of MTs growth (the length of MTs plus end growth events) increased significantly after EMT at both the cell interior and near the cell edge for all three cell lines (Fig. 3d and Supplementary Table S3); (iii) the angle between MT plus end growth tracks and cell radius decreased after EMT in all cases, except for interior of HaCaT cells (Fig. 3e and Supplementary Table S3).

In cancer cells, MT dynamics could be affected by the tubulin isoform profile, especially by the expression of βIII-tubulin isoform^29^. Therefore, we checked the expression of βIII-tubulin in all three cell lines before and after EMT and identified that it is upregulated after EMT (Supplementary Fig. S2e).

We conclude that after EMT while the overall density of growing MTs does not change, individual MTs become more dynamic and their growth is better aligned with cell radius.

### Actin cytoskeleton changes after EMT

Despite the profound alteration of cell morphology after EMT the difference in the organization of stress fibers was not obvious. Detailed analysis uncovered some changes in the actin fibers organization. Before EMT, prominent actin stress fibers were present in A549 and HaCaT cells, while in MCF-7 cells, the average number was low and some cells were lacking stress fibers. After EMT, all three cell lines expressed a significant amount of actin stress fibers crossing the cytoplasm.

**Figure 4:**
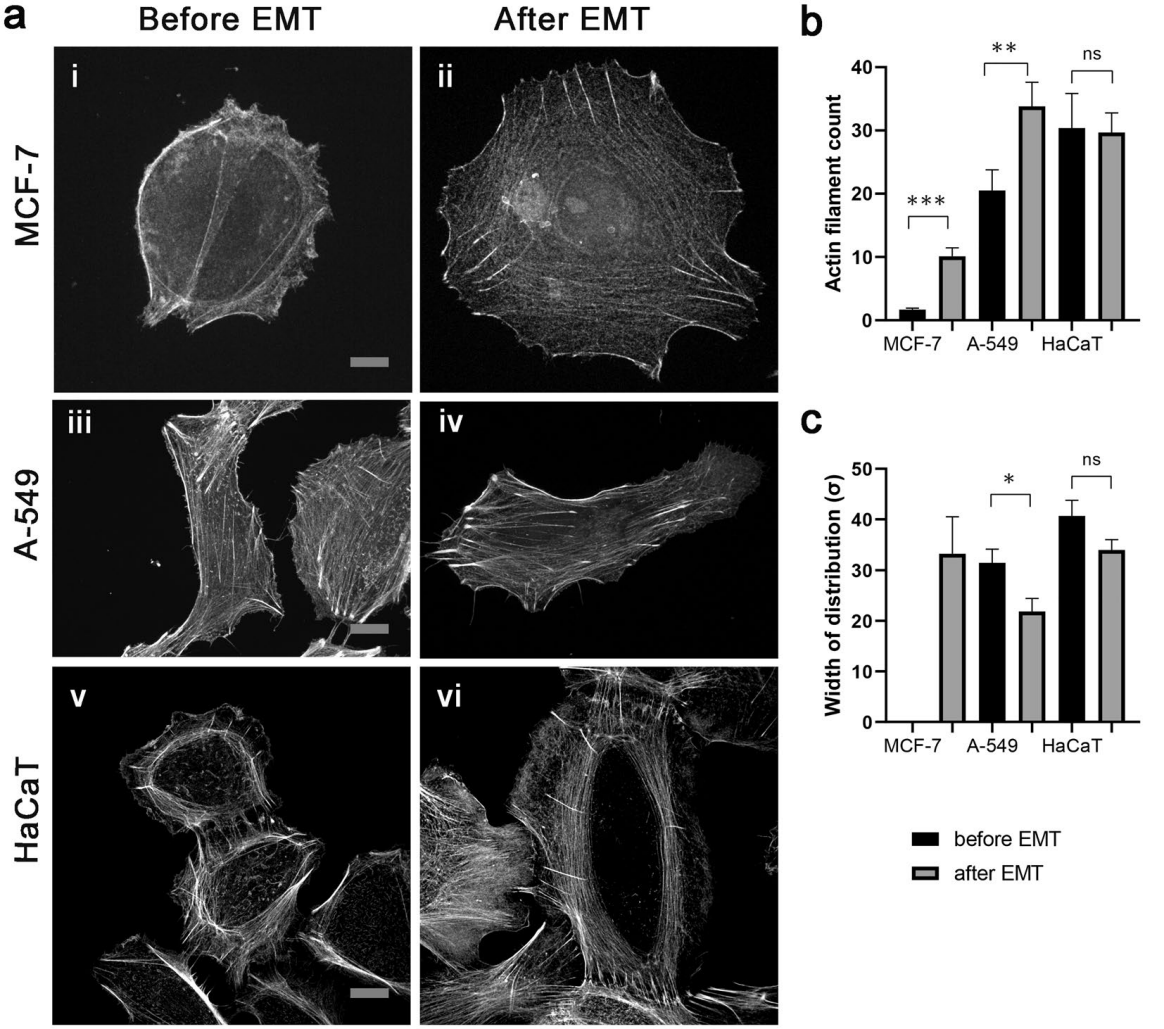
Fluorescence microscopy analysis of actin cytoskeleton rearrangements in MCF-7, A-549 and HaCaT cells after EMT. (**a**) Staining with Alexa Fluor 555-labeled phalloidin. (i, ii) MCF-7 cells before and after EMT. (iii, iv) A-549 cells before and after EMT. (v, vi) HaCaT cells before and after EMT. Scale bar 10 µm. (**b**) Actin stress fiber count in three different cell lines before and after EMT (MCF-7 before EMT, N = 28 cells; MCF-7 after EMT, N = 9 cells; A-549 before EMT, N = 27 cells; A-549 after EMT, N = 23 cells; HaCaT before EMT, N = 8 cells; HaCaT after EMT, N = 19 cells). (c) Angular distribution of actin filaments (σ) in cells before and after EMT (MCF-7 after EMT, N = 9 cells; A-549 before EMT, N = 19 cells; A-549 after EMT, N = 22 cells; HaCaT before EMT, N = 7 cells; HaCaT after EMT, N = 17 cells). Significance ***p < 0.001, **p < 0.01; *p < 0.05. The error bars represent mean ± SEM values.

The number of actin fibers in cells before and after EMT was calculated and a dramatic increase in actin fiber count in the cell bodies of MCF-7 cells after EMT was observed (Fig. 4b). The actin fibers were almost absent in cell bodies in MCF-7 cells before EMT (Fig. 4ai) and appeared after EMT (Fig. 4aii). In A-549 cells both before and after EMT, the actin filaments formed long stress fibers crossing the cytoplasm (Fig. 4aiii,iv). The amount of these fibers nearly doubled in A-549 after EMT (Fig. 4b). In HaCaT cells, prominent stress fibers were present before EMT and there was no increase after EMT (Fig. 4av,vi, Fig. 4b).

Next, the co-orientation of stress fibers before and after EMT was analyzed by determining the width of Gaussian distribution (Supplementary Fig. S10a and b) (Fig. 4c). A-549 and HaCaT cells after EMT experienced a shift towards more co-oriented fibers. The standard deviation of distribution after EMT in A-549 cells decreased by 32%. In HaCaT cells, it decreased by 15%, yet it was not statistically significant (Fig. 4c).

Since the number of stress fibers before EMT in MCF-7 cells was low, co-orientation was possible to determine there only after EMT (Fig. 4c) and it had the same variance as in HaCaT cells after EMT − σ = 33 ± 14.

### Focal adhesions in cells before and after EMT

**Figure 5:**
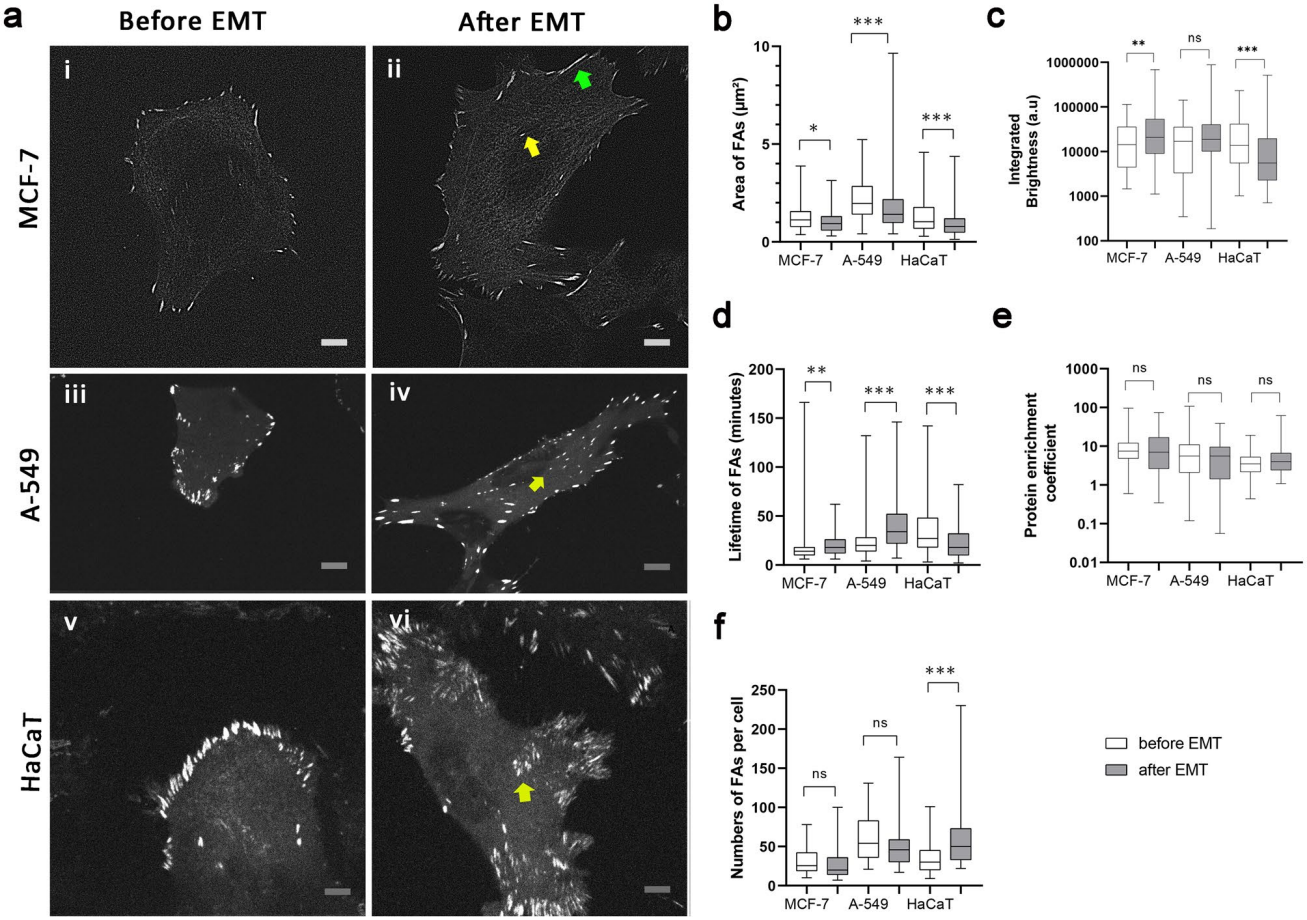
FAs distribution and their characteristics in three different cell lines: MCF-7, A-549 and HaCaT cells before and after EMT. (**a**) In MCF-7, A-549 and HaCaT cells before EMT, FAs were mostly localized at the cell periphery (i, iii, v), whilst after EMT a few FAs appeared in the cell body. (ii, iv, vi). Yellow arrowheads point to FAs in the inner parts of cells. The green arrowhead points FA with an elongated shape. Scale bar 10 µm. (**b**) Box plot showing the median (bold horizontal line), interquartile range (box) and maximum and minimum range (whiskers) of area of FAs, (**c**) of integrated brightness of FAs, (**d**) of lifetime of FAs, (**e**) of protein enrichment coefficient of FAs, (**f**) of the numbers of FAs per cell. The number of cells analyzed is indicated in Supplementary material Table S4. Significance ***p < 0.001, **p < 0.01; *p < 0.05. The error bars represent mean ± SEM values.

To characterize the morphology and dynamics of FAs in cells before and after EMT stable transfection of A-549 and HaCaT cells with *Ptag-RFP-vinculin* and transduction of MCF-7 cells with *Talin-RFP* was conducted. The FAs in cells before and after EMT were mostly oval-shaped, only a few were elongated (at the cell periphery) (Fig. 5aii). In cells before EMT FAs were mostly localized at the cell periphery (Fig. 5ai,iii,v), whilst after EMT FAs also appeared throughout the cytoplasm in A-549 and HaCaT cells (Fig. 5a,iv,vi) and to less extent in MCF-7 cells (Fig. 5aii).

After EMT in all three cell lines, the size of FAs slightly decreased: in MCF-7 cells—by 20%, in A-549 cells—by 14%, and in HaCaT cells—by 31% (Fig. 5b). Other changes in FA arrays after EMT were different in different cell lines.

The median integrated brightness of FAs in MCF-7 after EMT increased by 46%. In A-549 cells after EMT, it had a tendency to increase but not statistically significant. In HaCaT cells after EMT, the median integrated brightness on the opposite decreased by 60% (Fig. 5c).

The median lifetime of FAs in MCF-7 after EMT increased by 28%. In A-549 cells after EMT, it increased by 70%. However, in HaCaT cells the lifetime of FAs after EMT decreased by 33% (Fig. 5d).

The protein enrichment coefficient remained similar in all three cell lines (Fig. 5e). The number of FAs per cell did not change significantly in A549 and MCF-7 cells while nearly doubled in HaCaT cells after EMT (Fig. 5f) (Supplementary Table S4).

In summary, in MCF-7 and A-549 cells, upregulation of FAs was achieved through the increase in the lifetime of individual FAs and increased integrated brightness. In contrast, in HaCaT cells—the lifetime of individual FAs and integrated brightness of FAs decreased, whilst the number of FAs per cell increased.

## Discussion

In this study, we intended to analyze the consequences of EMT for the cytoskeleton organization using three cell lines of epithelial origin, A-549, HaCaT and MCF-7 cells. For A-549 and HaCaT cells we induced EMT by using TGF-β1, while MCF-7 cells were induced through the inserted inducible Tet-On system, allowing us to validate the activation of EMT at individual cell level (by green fluorescent nucleus staining). All three cell lines experienced morphological changes after EMT consistent with the previously described^26,27,30^: cells increased in size, became more spread out on the substrate and formed larger lamellae. After EMT, MCF-7 and HaCaT cells became spread out, while A-549 cells became spread out and elongated. It is important to notice that the variability of morphological characteristics after EMT did not change dramatically allowing us to suggest that the majority of the cells treated underwent transition in our experiments.

Along with morphological changes, we observed changes in microtubule spatial organization and dynamics, actin cytoskeleton and focal adhesion patterns. Each component of the cytoskeleton was examined using multiple parameters and the following alterations were identified in the cytoskeleton:

### Microtubules in EMT

Our data shows that cells after EMT have a larger area covered by MTs in all three cell lines and the density of MTs near the cell edges decreases. MTs became more dynamic and at the same time have distal segments oriented perpendicularly towards the plasma membrane. These changes could be explained by the stimulation of MT growth in the cell interior^31,32^.

Alteration of growth of MTs, in turn, could be explained by increased expression of β-tubulin III after EMT described elsewhere^33–36^ and confirmed in our study (Supplementary Fig. S2e). However, the effect of elevated expression of β-tubulin III on MT dynamics remained uncertain and the results of direct measurements show that increased dynamics of MTs containing β-tubulin III is observed in some cancer cells (A435)^29,37^ while it is impeded in other types cells and in vitro^38,39^. We found that not only MT growth is stimulated by EMT but also the spatial organization of the MT array is “improved”—it becomes more radial, especially at cell edges. We suppose that MT growth after EMT becomes restricted by a plasma membrane. This can happen as a result of elevated expression of MT plus-end binding proteins described elsewhere^40,41^. Also, such behavior is similar to that of short-living MTs observed at the axon growth cone^32^, or in fish melanophores after adrenalin stimulation^42,43^. Long-living MTs at the cell edge usually bend and continue growing along the plasma membrane^44,45^. The mechanism of this regulation requires further elucidation. In addition, we suggest that the limitation of the MT plus end growth at the plasma membrane allows the cell to keep a large pool of soluble tubulin and has a funneling effect for MT growth^46^. This effect is well known for actin at the cell edge^47,48^ and we assume that similar regulation facilitates the formation of the extended radial MT array. Moreover, the limitation of the MT plus ends growth at the cell edges along with stimulated growth in the cytoplasm may contribute to leading edge formation and directional and persistent cell motility, as shown in motile fibroblasts^49^, and might be critical for tumor cells invasion and metastases into distal organs.

### Actin cytoskeleton

Our study reveals that all three cell lines underwent a reorganization of their actin cytoskeleton after EMT. The number of thick actin fibers increased in A549 and MCF-7 cells, while in HaCaT cells stress fibers were only better co-aligned.

Cells undergoing EMT may have a higher potential for migration and invasion since stress fibers can regulate multiple cellular functions in migrating cells, including the maturation of integrin-based adhesions like focal adhesions^14^. Stress fibers can also interact with myosin II^50,51^ and generate force, which promotes cell movement by the contraction of actin fibers. Stress fibers also assist in establishing the front-rear polarity axis which is essential in EMT^14^. However, some studies suggest that stress fibers may indicate a reduction in the turnover of actin filaments, leading to more stable actin fibers and decreasing cell motility and cancer cell invasion^52^. We assume that the relationship between stress fibers and cell motility in fibroblasts and cancer cells might be bi-phasic: lack or low number of stress fibers does not allow efficient organization of front-rear polarity, while excessive stress fibers along with large FAs will inhibit cell motility. The moderate increase in the number of actin fibers that we observed might be necessary for stimulating cell migration.

Therefore, we along with others^53^ suggest that when cells after EMT exhibit more co-aligned thick actin fibers crossing the cytoplasm, they become polarized, with intensive protrusions forming on the leading edge. Improved co-alignment of actin fibers could limit the formation of random protrusions and instead facilitate the formation of front-rear polarity. Besides, the interaction between radial microtubules and actin fibers oriented towards the leading edge can facilitate the delivery of signaling molecules or membrane components necessary for lamellipodial protrusions^54^.

### FAs during EMT

The overall pattern of FAs and the dynamics of these structures changed after EMT in all three cell lines studied. We observed two morphological features of FAs changing during EMT: the average area of FAs decreased and FAs appeared more frequently in cell bodies. This is in accord with the previous description of the behavior of FAs in epithelial cells undergoing EMT^55,56^. The localization of FAs more centrally after EMT may be associated with increased cell migration since as cells move FAs are apparently translocated towards the cell center^57^. However, our findings do not support the assumption that the size of FAs positively correlates with cell speed^1^. This conclusion was made on fibroblasts, and we assume that the functional role of FAs in motile cancer (epithelial) cells and fibroblasts might be different.

Following the assumption that the size of FAs may be in a biphasic relationship with cell migration speed^58^ and keeping in mind that the motility of cells after EMT increases^59^, we can predict the threshold upon which cell speed does not correlate with FAs size in cancer cells. In cell lines after EMT the median area of FAs decreased to less than 1 µm^2^ for MCF-7 and HaCaT cells, and < 1.5 µm^2^ for A-549 cells. Thus, we suggest that the threshold size of FAs above which cell speed does not positively correlate with cell motility in cancer cells is around 1 µm^2^.

We observed significant changes in MT dynamics in all three cell lines after EMT, however the changes in the rate of FAs turnover (lifetime of FAs) for MCF-7, A-549 and HaCaT cells after EMT were opposite. Therefore we cannot conclude that MT dynamics is directly associated with FAs turnover and further studies of the relationship between these parameters are necessary.

## Conclusion

We conclude that the following parameters of the cytoskeleton characterize EMT: increased cell area covered by MTs and F-actin, radial microtubule organization, increased MTs’ plus end growth, decreased angle of growth of MTs’ plus ends trajectories in respect to cell radius, increased co-alignment of actin stress fibers, and decreased size of FAs. Other changes in the cytoskeleton appear to be cell line-specific.

Further studies on cytoskeleton organization after EMT in different cell cultures are necessary to elucidate the mechanisms underlying cytoskeleton alterations, determine if these alterations are consistent across a wide range of cell lines, and explore the potential relationship between increased microtubule dynamics, increased length of MTs’ plus end growth events, and focal adhesion distribution.

Actin stress fibers and reorganized microtubules can generate several distinct force-inducing mechanisms in a cell together to stimulate cell migration. However, the molecular mechanisms underlying these modifications remain largely unknown. The following questions are evident from our study: (i) Whether elevated expression of βIII-tubulin per se affect the organization of MTs and actin cytoskeleton? (ii) Whether a pharmacological cocktail for arresting actin dynamics and microtubule dynamics together may inhibit cell motility during EMT? (iii) What mechanisms are involved in the regulation of FA dynamics in cancer and non-cancer motile cells?

## Materials and methods

### EMT Induction and cell culture

We induced EMT in lung carcinoma A-549 and in immortalized human keratinocyte HaCaT cells obtained from American Type Culture Collection (ATCC, VA, USA) using TGF-β1 at the concentration of 5 ng/mL (added every day for 3 days) and in modified human breast adenocarcinoma cells MCF-7 (gift from Prof. E. Tulchinsky). The MCF-7 cells have inserted an inducible Tet-On system with ZEB1-GFP fusion construct that is activated by the addition of doxycycline (10 µg/mL daily for 48–72 h) and leads to the expression of the master regulator of EMT, Zeb-1^11^ that could be determined by green fluorescence in the nucleus^26^. Cells were grown in Dulbecco’s modified Eagle medium (DMEM) (Thermo Fisher Scientific, Cat. # 11965092) supplemented with 10% fetal bovine serum (FBS) (Thermo Fisher Scientific, Cat. # 10100147), 4–8 mM of l-glutamine (Sigma, Cat. # G7513) and antibiotics penicillin–streptomycin (Sigma-Aldrich, Cat. # P4333) in a humidified incubator containing 5% CO_2_ at 37 °C.

### Evaluation of morphological changes

In order to describe morphological changes in MCF-7, A-549 and HaCaT cells after EMT, we did area and aspect ratio measurements using Fiji ImageJ software. Binary images were created by thresholding the images to remove background noise. The area measurements were performed on the binary images using the 'Wand (tracing) tool’ in Fiji ImageJ (Supplementary Fig. S11). The aspect ratio of cells was calculated based on their major and minor axes. The major axis refers to the longest axis inside the cell, while the minor axis is the perpendicular axis to the major axis and is located in the middle of the cell when the cell shape resembles an oval (Supplementary Fig.S12 a). In cases where the cell shape is different from an oval, the major axis is still chosen as the longest axis inside the cell, while the minor axis is arbitrarily chosen to be perpendicular to the major axis and maximally located close to the relative cell center (Supplementary Fig.S12b). To calculate the aspect ratio, the length of the major axis was divided by the length of the minor axis for each of the 100 randomly selected cells.

### Microtubule dynamic measurements

Transient transfection of MCF-7, A-549 and HaCaT cells was carried out using X-treme GENE HP DNA Transfection Reagent (Roche, Switzerland) according to the manufacturer’s instructions. Briefly, MCF-7 A-549 or HaCaT cells were cultured in eight-chambered cover glass plates in DMEM supplemented with 5% FBS in a humidified incubator containing 5% CO_2_ at 37 °C for 24 or 48 h with or without the addition of EMT stimulator. Then, cells were transfected with 1 μg of EB-3-RFP plasmid DNA^60^ by X-treme GENE HP DNA transfection reagent in PBS. Transfection was carried out in parallel with EMT induction. The optimal ratio of the plasmid to transfection agent was 1:3. At 24 h after transfection, the medium was replaced by a CO2-independent medium, and then cells were visualized by microscopy. Then, using Fiji Image J software, we analyzed microtubule dynamics by manual tracking (Fig. 3a). Thus, we determined the speed, time and track length of individual plus ends of microtubules in cells before and after EMT.

### The measurement of the length of MTs plus end growth events, angle of growth and the density of MTs distribution within the cell

To quantify the length of MTs growth events, angle of growth with respect to cell radius and the density of MT distribution in cells before and after EMT, we constructed maximum intensity projections (MIPs) of 20 s (10 frames of video) for EB-3-RFP tracks. To identify the cell interior and cell edge parts, we divided the cell area into three regions: (1) the centrosome area, which is defined as a distance of ≤ 4.75 µm from the centrosome. We did not analyze microtubules in this area because of their high density. (2) The cell interior, is defined as a distance > 4.75 µm from the centrosome and > 2.8 µm from the cell margin. (3) The cell marginal area, which is defined as a distance ≤ 2.8 µm from the cell margin. Then, we measured and identified the aforementioned parameters within each part (Supplementary Fig.S9a, b). The cell border was defined as a region around the cell periphery where MTs are densely packed (Supplementary Fig.S9c,d).

### Transduction with Talin-RFP and transient transfection with Ptag-RFP-Vinculin and the use of A-549 and HaCaT cellular lines with stable transfection of vinculin to visualize focal adhesions

Cells were labeled utilizing Cell Light Talin-RFP, BacMam 2.0 (Invitrogen), following the manufacturer’s instructions. The Cell Light reagent was added to cells at about 30% confluence and incubated for 24 h at 37 °C in 5% CO_2_. Transfection was carried out in parallel with EMT induction. After talin-RFP, expression was confirmed using fluorescent microscopy.

Transient transfection of MCF-7, A-549 and HaCaT with Ptag-RFP-vinculin vector (Eurogen, Russia, Cat. # FP372) was carried out using X-treme GENE HP DNA transfection reagent (Roche, Switzerland) according to manufacturer’s instructions.

A-549 and HaCaT cells with stable transfection of vinculin (gift from A. Saidova) were incubated for 3 days in the presence of EMT inducer (5 ng/mL TGF- β1) and analyzed under a microscope.

### Immunofluorescent analysis

Cells were fixed with 1% glutaraldehyde and microtubules were immunofluorescent labeled using Anti-α-Tubulin (T9026) (Sigma-Aldrich) followed by secondary antibodies Alexa Fluor 488 Goat Anti-Mouse SFX Kit (Cat. # A31620) (Invitrogen) or using Alexa Fluor 594 Anti-alpha Tubulin antibody [EP1332Y]—Microtubule Marker (ab202272) (Abcam). F-actin was immunofluorescent labeled using Alexa Fluor 555 Phalloidin (Cat. # A34055) (Invitrogen). DAPI (Sigma-Aldrich, St. Louis, MO, USA) was used for nucleus visualization.

### Microscopic image acquisition

The microscopic observations were performed using the Zeiss Cell Observer and the Zeiss Cell Observer with Spinning Disk Confocal Unit (CSU-W1 Yokogawa). The Zeiss Cell Observer microscope was controlled using the ZEN software. The Zeiss Cell Observer Spinning Disk Confocal microscope was controlled using MetaMorph software. Both microscopes were equipped with a heating incubator chamber. Zeiss Cell Observer was equipped with an ORCA-Flash 4.0 V2 camera (Hamamatsu Photonics, Hamamatsu, Japan). Spinning Disk Confocal microscope was equipped with a Photometrics PRIME-95B camera. Brightfield and fluorescent time-lapse microscopy were used to capture images and recordings. The fluorescence microscopy observations were performed using Plan–Apochromat × 63/1.4 (oil immersion) and × 100/1.49 (oil immersion) objectives, LED light source Colibri 2. The following filter cube sets were used: filter set 49 (excitation: G 365, beam splitter: FT 395 and emission: BP 445/50), filter set 38 HE (excitation: BP 470/40 (HE), a beam splitter: FT 495 (HE), and emission: BP 525/50 (HE)), and filter set 43 HE (excitation: BP 550/25 (HE), a beam splitter: FT 570 (HE), and emission: BP 605/70 (HE)). Images on a spinning disk microscope were collected using the following laser wavelengths: 355 nm, 488 nm and 561 nm with corresponding filter sets. Time-lapse series recordings for microtubule dynamic measurements were made with intervals of 2 s during 100 cycles. Duration of the observation of focal adhesions turnover was observed during 4–6 h with intervals of 2 min between frames.

### Actin fiber measurements

Widefield immunofluorescent images (visualized via Alexa Fluor 555-phalloidin) of actin fibers were manually traced via a custom MATLAB script. The resulting actin fiber positions were further analyzed via another custom MATLAB script (code is included in supplementary materials). Briefly, this analyzer assesses the number of actin fibers (beyond the minimal length threshold of 40 pixels/4 µm) and their relative direction (Supplementary Fig. S10c,d,e). The angular distribution of fiber directions is plotted as a histogram and fitted with a normal distribution (Supplementary Fig.S10a,b). Sigma of the resulting normal distribution is used as a measure of directional similarity, with smaller sigma corresponding to a more narrow angular distribution of more unidirectional actin fibers. Only cells with more than 10 sufficiently long fibers were included in statistics for sigma distribution, as smaller groups of fibers cannot be robustly assessed for co-directionality.

### Measurement of area, integrated brightness, protein enrichment coefficient and lifetime of focal adhesions

To measure area, integrated brightness and protein enrichment coefficient of FAs around 10 the biggest and the brightest focal adhesions where borders clearly can be seen was chosen in each analyzed cell. The measurements were done using Fiji Image J and the commands in the supplementary material (Supplementary Fig. S13). To measure the lifetime of FAs only focal adhesions that were assembled and disassembled during the time-lapse film recordings were analyzed. Lifetime of FA = quantity of frames ^ interval of time-lapse film (minutes).

### Western blot detection

Cells were lysed in RIPA buffer for 30 min. Protein concentration was measured using Bradford assay (ab102535; Abcam). Protein lysates were resolved in 12% SDS‐PAGE gel and transferred to 0.4 μm Immobilon-P PVDF membranes (Millipore), blocked with 3% BSA, 1X TBS, 0.1% Tween‐20 for 150 min at RT. Membranes were incubated with the rabbit anti-E-cadherin (5:10,000; ab 40772; Abcam), anti N-cadherin (5:10,000; ab 76011; Abcam), and anti-SLUG primary antibodies(1:1000; PRS3957; Sigma-Aldrich), anti-GAPDH (1:10,000; ab181602; Abcam) at + 4 °C overnight in 0.5% non-fat dry milk in 1X TBS, 0.1% Tween‐20. After washing trice, membranes were incubated with the horseradish peroxidase-conjugated goat anti-rabbit secondary antibodies (1:2000; ab 6721; Abcam) for 1 h. at RT. Membranes were washed again, and a Novex HRP Chromogenic substrate (TMB) (Invitrogen) or Immobilon ECL Ultra Western HRP Substrate (Millipore) was used to visualize the protein blots.

### RNA extraction and reverse transcription

RNA was extracted from frozen cell samples using TRIzol Reagent (Invitrogen, Cat. #15596026) according to the manufacturer’s protocol. The reverse transcription was performed as described elsewhere^61^.

### RT qPCR

Real-time qPCR experiments were performed on CFX96 Touch cycler (Bio-Rad, Hercules, CA, USA). Primer details are provided in Supplementary material (Table S5). The reaction volume was 20 μl and consisted of 10 μl SsoAdvanced Universal SYBR Green Supermix, 1 μl of forward and 1 μl of reverse primers, 2 μl of cDNA and 6 μl nuclease-free water. The reaction protocol included denaturation at 95 °C followed by 39 amplification cycles and further melting curve analysis. All samples were processed in duplicate.

### Statistical analysis

The statistical analysis and data plotting were performed using Graph Pad Prism (Graph Pad Software, version 5, San Diego, CA, USA) and a nonparametric Mann–Whitney *U* test or parametric t-test with Welch correction. Significance ***p < 0.001, **p < 0.01; *p < 0.05. The error bars represent mean ± SEM values.

### Supplementary Information

Supplementary Figure S1.

Supplementary Figure S2.

Supplementary Figure S3.

Supplementary Figure S4.

Supplementary Figure S5.

Supplementary Figure S6.

Supplementary Figure S7.

Supplementary Figure S8.

Supplementary Figure S9.

Supplementary Figure S10.

Supplementary Figure S11.

Supplementary Figure S12.

Supplementary Figure S13.

Supplementary Video 1.

Supplementary Video 2.

Supplementary Video 3.

Supplementary Video 4.

Supplementary Video 5.

Supplementary Video 6.

Supplementary Video 7.

Supplementary Video 8.

Supplementary Video 9.

Supplementary Video 10.

Supplementary Video 11.

Supplementary Video 12.

Supplementary Information 1.

Supplementary Information 2.

Supplementary Information 3.

Supplementary Table S1.

Supplementary Table S2.

Supplementary Table S3.

Supplementary Table S4.

Supplementary Table S5.

Supplementary Legends.

## Data Availability

All data generated or analyzed during this study are included in this article and its supplementary information files.
