# Supplementary material for: Morphological and cytoskeleton changes in cells after EMT
Source: Sci Rep. 2023 Dec 13;13:22164. doi: 10.1038/s41598-023-48279-y (PMC10719275; doi:10.1038/s41598-023-48279-y)
Supplement: Supplementary file 1 — Supplementary Figure S1. [file 41598_2023_48279_MOESM1_ESM.docx]

**Supplementary Materials:**

**
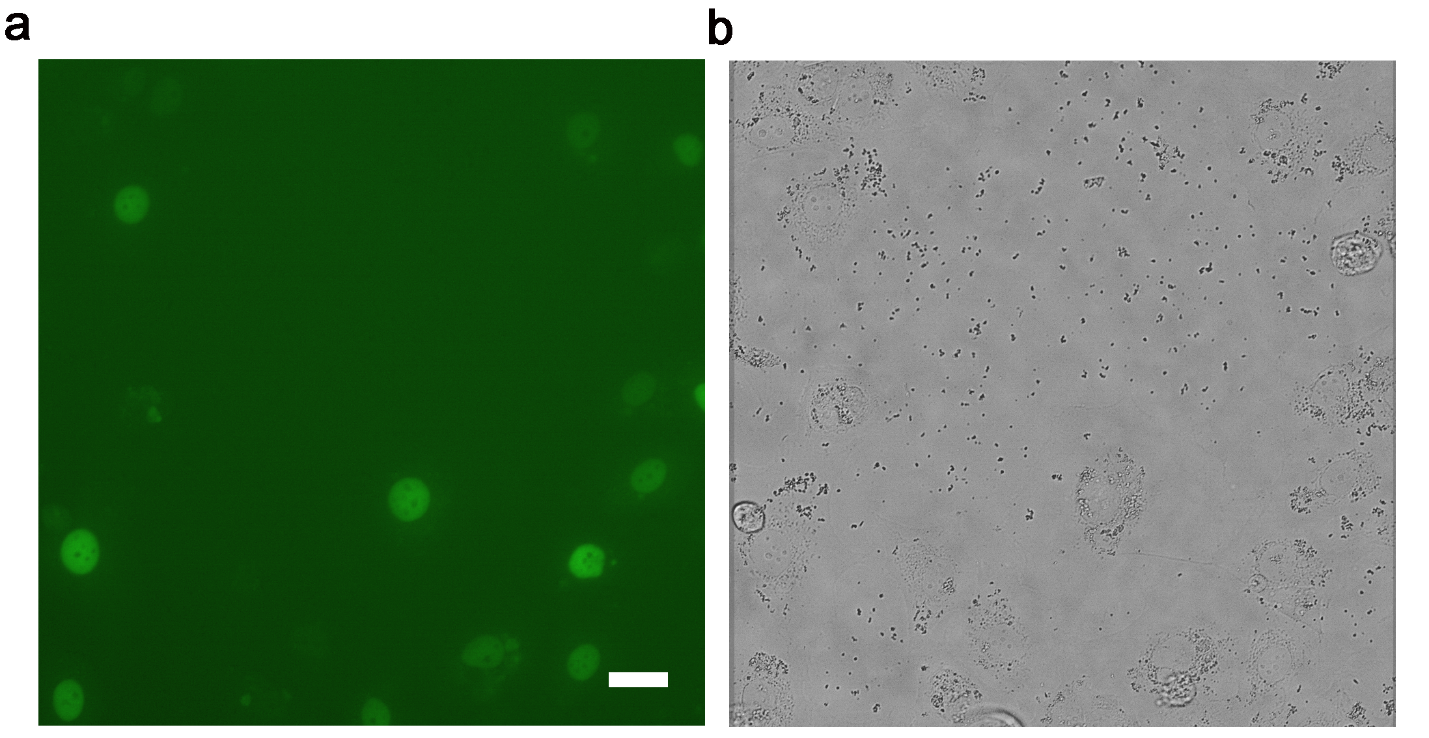
**

**Figure S1.** MCF-7 cells after EMT induction. (a) GFP channel. MCF-7 cells after doxycycline treatment. The treatment by doxycycline enables to initiation of the expression of GFP-tagged ZEB-1 protein. The efficiency of EMT launched process in the cells was visually seen by the green fluorescent nucleus staining. (b) Widefield. Scale bar 10µm.
