# Supplementary material for: Morphological and cytoskeleton changes in cells after EMT
Source: Sci Rep. 2023 Dec 13;13:22164. doi: 10.1038/s41598-023-48279-y (PMC10719275; doi:10.1038/s41598-023-48279-y)
Supplement: Supplementary file 2 — Supplementary Figure S2. [file 41598_2023_48279_MOESM2_ESM.docx]

**
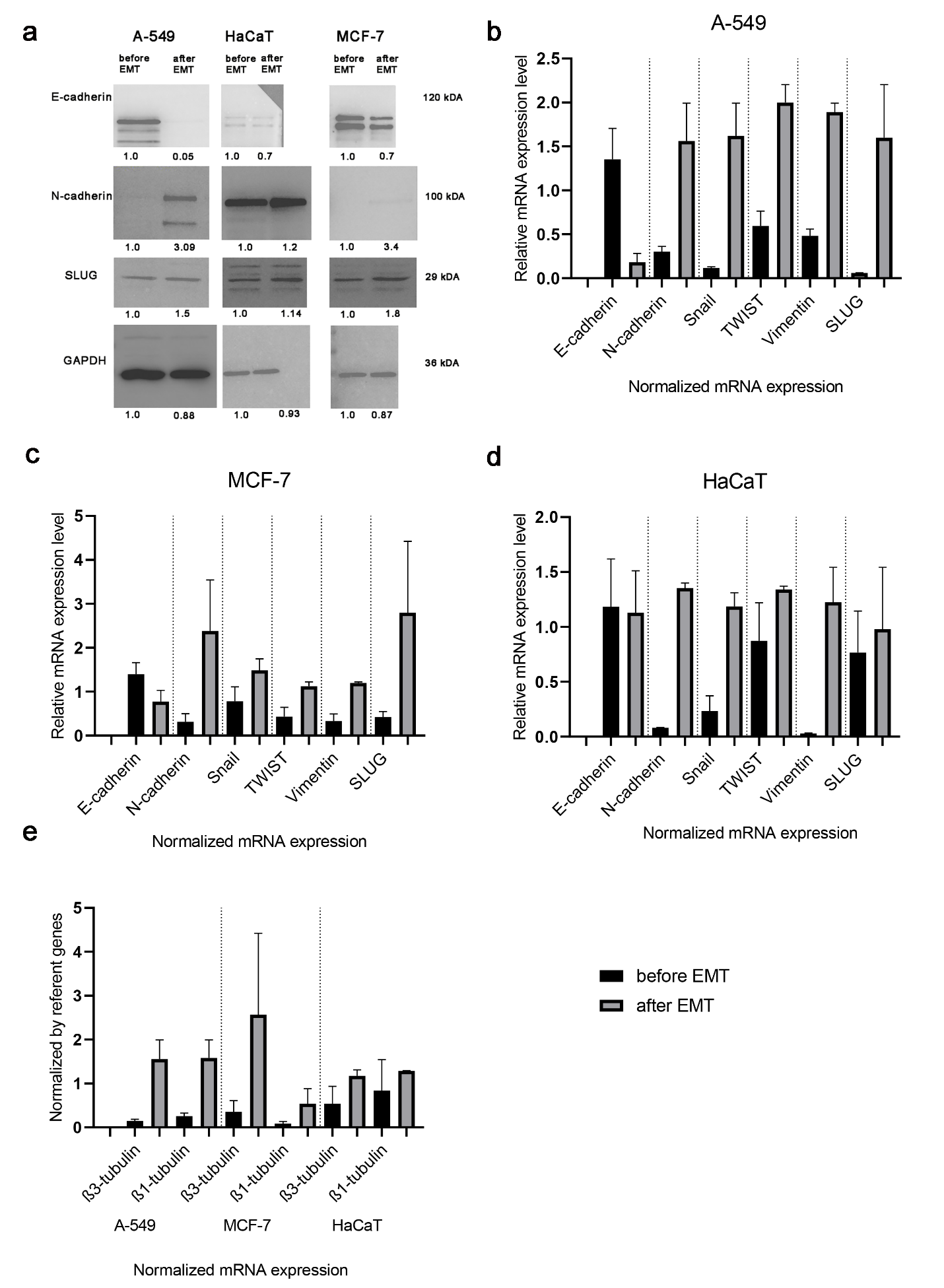
**

**Figure S2.** Western blot and RT-PCR analysis of EMT-related molecules. (a) Cropped blots. Epithelial phenotype marker, E-cadherin, had been highly expressed in A-549 cells before EMT (untreated cells) and downregulated after TGF-β1 treatment, while mesenchymal phenotype-associated proteins, N-cadherin and Slug were all upregulated in transformed A-549 cells. Alternatively, E-cadherin expression in MCF-7 and HaCaT cells before and after EMT remained at a similar level, and only expression of N-cadherin and Slug for MCF-7 cells after EMT and Slug for HaCaT cells after EMT were slightly upregulated. The samples for each cell line were derived from the same experiment and the gels/blots were processed in parallel. (b) Relative mRNA expression of EMT-associated genes using RT-PCR after EMT induction in MCF-7, (c) A-549 and (d) HaCaT cells. (e) Relative mRNA expression of ß3 and ß1- tubulin in A-549, MCF-7 and HaCaT cells. Real-time quantitative PCR data was normalized to UBC and HPRT1 reference genes.
