# Supplementary material for: Morphological and cytoskeleton changes in cells after EMT
Source: Sci Rep. 2023 Dec 13;13:22164. doi: 10.1038/s41598-023-48279-y (PMC10719275; doi:10.1038/s41598-023-48279-y)
Supplement: Supplementary file 3 — Supplementary Figure S3. [file 41598_2023_48279_MOESM3_ESM.docx]

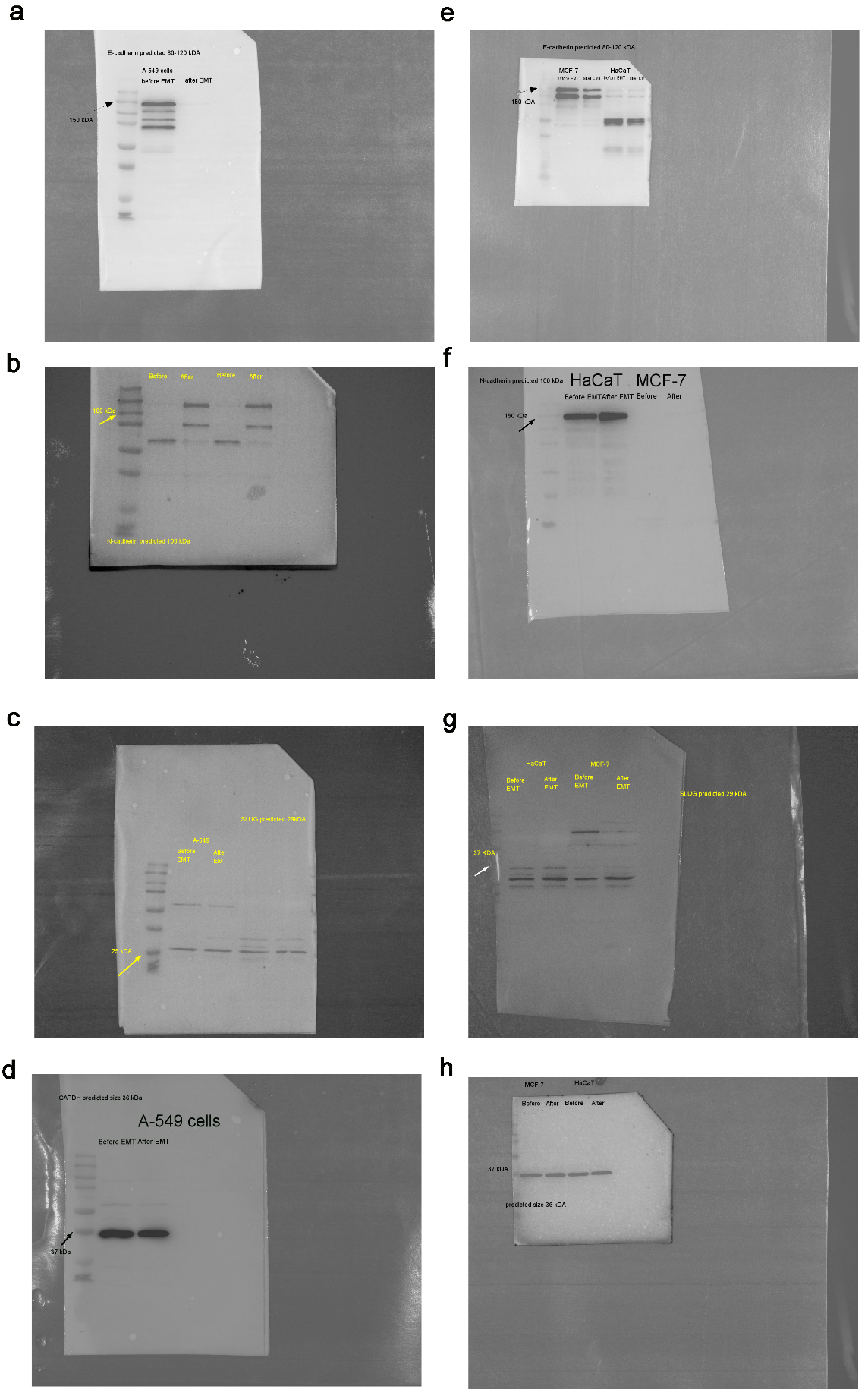


**Figure S3.** Original gels/blots. (a) E-cadherin expression in A-549 cells before and after EMT. (b)N-cadherin expression in A-549 cells before and after EMT. (c)Slug expression in A-549 cells before and after EMT. (d)GAPDH expression in A-549 cells before and after EMT. (e)E-cadherin expression in MCF-7 and HaCaT cells before and after EMT.(f) N-cadherin expression in MCF-7 and HaCaT cells before and after EMT.(g) Slug expression in MCF-7 and HaCaT cells before and after EMT.(h)GAPDH expression in A-549 cells before and after EMT.
