# Supplementary material for: Morphological and cytoskeleton changes in cells after EMT
Source: Sci Rep. 2023 Dec 13;13:22164. doi: 10.1038/s41598-023-48279-y (PMC10719275; doi:10.1038/s41598-023-48279-y)
Supplement: Supplementary file 4 — Supplementary Figure S4. [file 41598_2023_48279_MOESM4_ESM.docx]

**
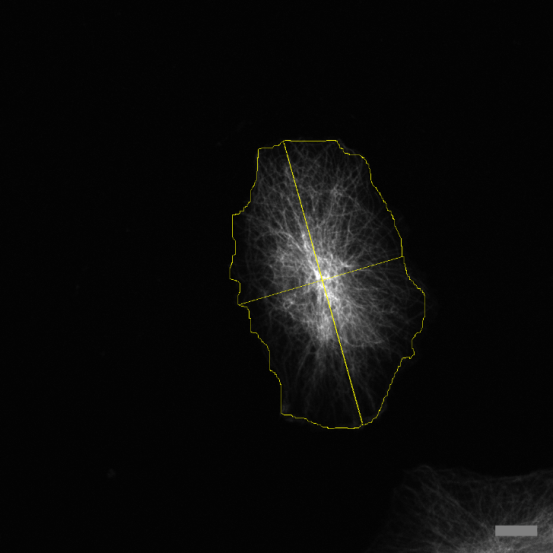
**

**Figure S4.** Criteria “microtubule-covered area”. Scale bar 10µm.

To measure microtubule-covered area in Fiji ImageJ following procedure was chosen:

Open the image of interest in ImageJ.

Select the "Freehand Selection" tool from the toolbar.

Draw a boundary around the microtubule-covered area of the cell

Choose Analyze >Tools> ROI Manager >Add>Measure
