# Supplementary material for: Morphological and cytoskeleton changes in cells after EMT
Source: Sci Rep. 2023 Dec 13;13:22164. doi: 10.1038/s41598-023-48279-y (PMC10719275; doi:10.1038/s41598-023-48279-y)
Supplement: Supplementary file 5 — Supplementary Figure S5. [file 41598_2023_48279_MOESM5_ESM.docx]

**
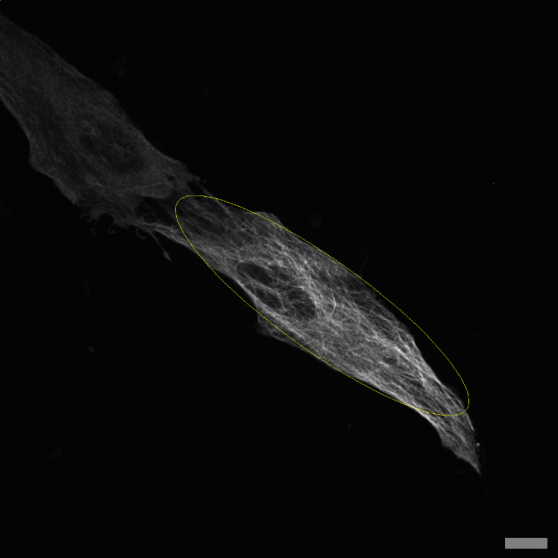
**

**Figure S5.** Criteria“circularity”. Circularity is a measure of how closely the shape of a cell area covered with MTs approaches a circle.

To measure the circularity of the cell of interest, the following procedure was used:

Open the image in Fiji ImageJ.

Select the "Oval" tool from the toolbar.

Choose "elliptical selections”

Rotate the ellipse so that the main cell part fits the figure

Choose Analyze >Tools> ROI Manager >Add>Measure

Circularity measurements will be displayed in the "Results" window, where 1 represents a perfect circle and values closer to 0 represent increasingly elongated shapes. Scale bar 10µm.
