# Supplementary material for: Morphological and cytoskeleton changes in cells after EMT
Source: Sci Rep. 2023 Dec 13;13:22164. doi: 10.1038/s41598-023-48279-y (PMC10719275; doi:10.1038/s41598-023-48279-y)
Supplement: Supplementary file 6 — Supplementary Figure S6. [file 41598_2023_48279_MOESM6_ESM.docx]

**
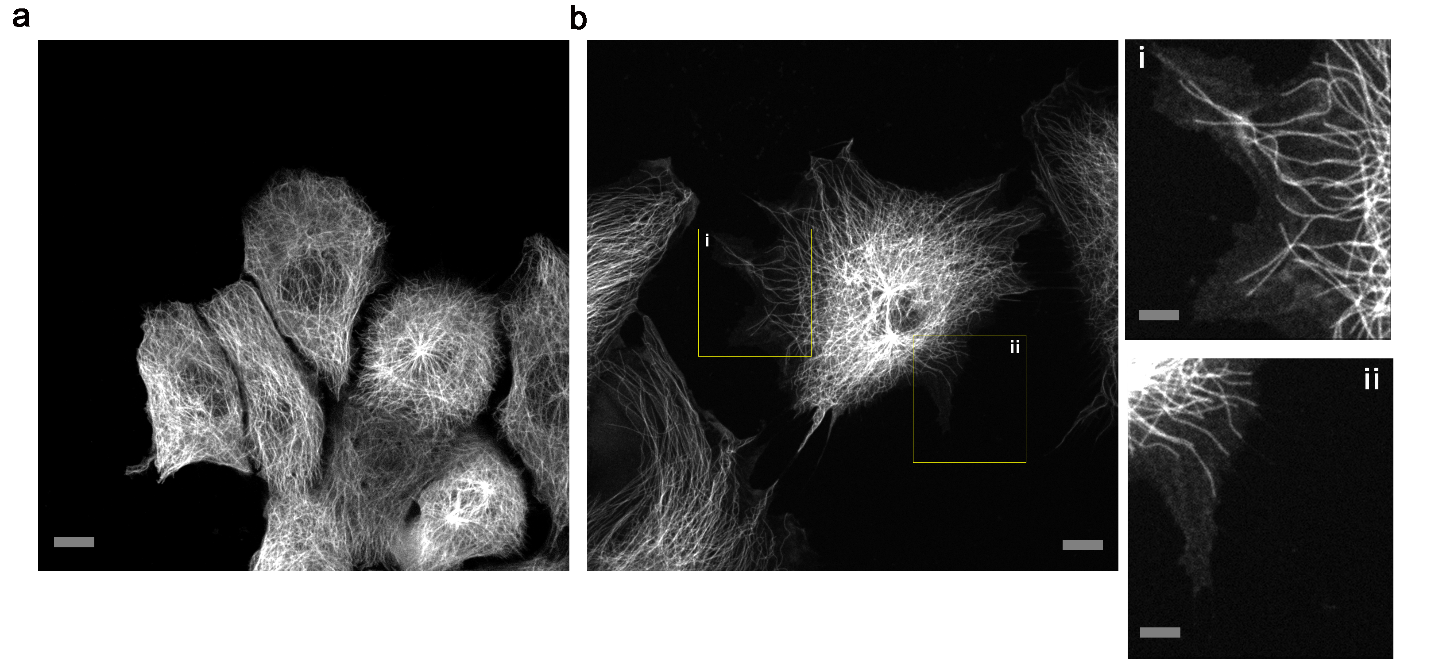
**

**Figure S6.** Criteria “individual MTs at cell edge”. “Individual” microtubules are generally defined as microtubules that are clearly visible for 3 µm or more of their length and form a region with at least five such microtubules nearby facing away from the cell interior without intersecting with other microtubules in that region. Cell edges facing directly into another cell (another cell is within 5 µm) are excluded from this analysis. (a) Example of a cell with no visible individual microtubules at the cell edge. (b) Example of a cell that has individual microtubules at cell edge (i and ii) Scaled region. Scale bar 10µm.
