# Supplementary material for: Morphological and cytoskeleton changes in cells after EMT
Source: Sci Rep. 2023 Dec 13;13:22164. doi: 10.1038/s41598-023-48279-y (PMC10719275; doi:10.1038/s41598-023-48279-y)
Supplement: Supplementary file 7 — Supplementary Figure S7. [file 41598_2023_48279_MOESM7_ESM.docx]

**
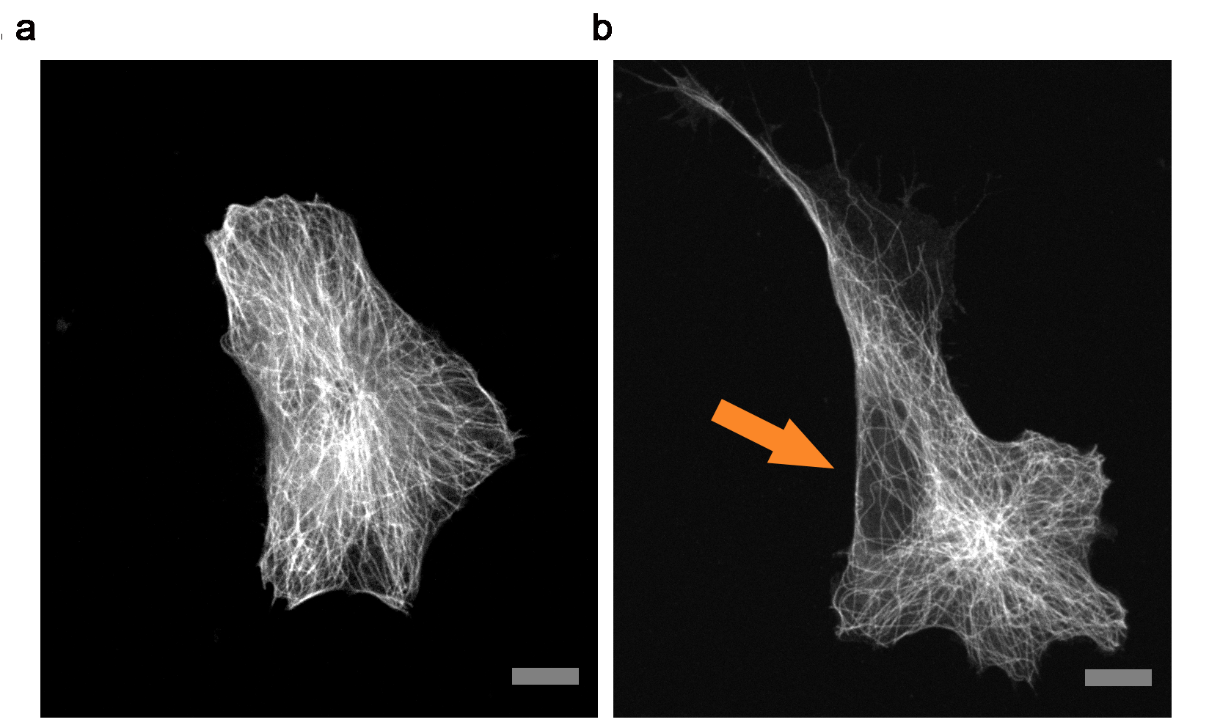
**

**Figure S7.** Criteria “nucleus coverage by microtubules”. We evaluated nucleus coverage with binary “yes” or “no” criteria. When the nucleus edge could not be easily distinguished from the surrounding cytoplasm due to the high microtubule density in front of it, the nucleus was considered covered. (a) An example of a cell with a nucleus completely obscured by microtubules. (b) An example of a cell with a nucleus area (red arrow) could easily distinguished from the surrounding cytoplasm. Scale bar 10µm.
