# Supplementary material for: Morphological and cytoskeleton changes in cells after EMT
Source: Sci Rep. 2023 Dec 13;13:22164. doi: 10.1038/s41598-023-48279-y (PMC10719275; doi:10.1038/s41598-023-48279-y)
Supplement: Supplementary file 8 — Supplementary Figure S8. [file 41598_2023_48279_MOESM8_ESM.docx]

**
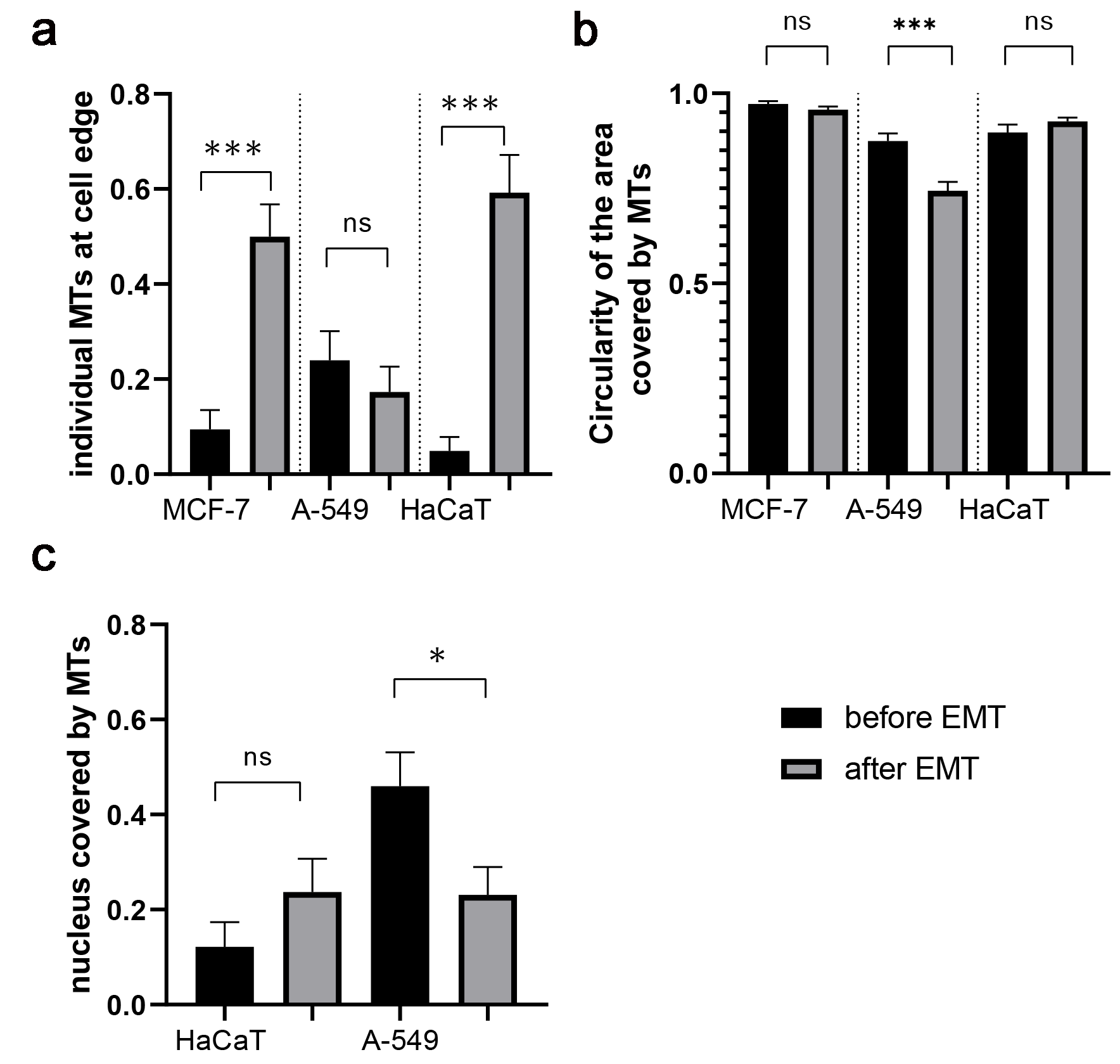
**

**Figure S8.** Three characteristics of MTs in cells before and after EMT. (a) The presence of individual microtubules resolvable at the cell edge (as defined in S6). MCF-7 cells before EMT (N=53 cells)/ after EMT (N=56 cells); A-549 cells before EMT (N=50 cells)/after EMT (N=52 cells); HaCaT cells before EMT (N=41 cells)/after EMT (N=38 cells). (b) Circularity of the area covered by microtubules (as defined in S5). MCF-7 cells before EMT (N=53 cells)/ after EMT (N=56 cells); A-549 cells before EMT (N=50 cells)/after EMT (N=52 cells); HaCaT cells before EMT (N=40 cells)/after EMT (N=38 cells). (c) The coverage of the nucleus by microtubules (as defined in S7). A-549 cells before EMT (N=50 cells)/after EMT (N=51 cells); HaCaT cells before EMT (N=41 cells)/after EMT (N=38 cells). Data for MCF-7 was not shown since GFP expressed in nuclei after EMT and it was spectrally interfering with goat anti-Mouse IgG, Alexa Fluor 488 (Cat. # A-11029) antibodies used for immunofluorescence. Significance *** - p <0.001, * - p <0.05. The error bars represent mean ± SEM values.
