# Supplementary material for: Morphological and cytoskeleton changes in cells after EMT
Source: Sci Rep. 2023 Dec 13;13:22164. doi: 10.1038/s41598-023-48279-y (PMC10719275; doi:10.1038/s41598-023-48279-y)
Supplement: Supplementary file 9 — Supplementary Figure S9. [file 41598_2023_48279_MOESM9_ESM.docx]

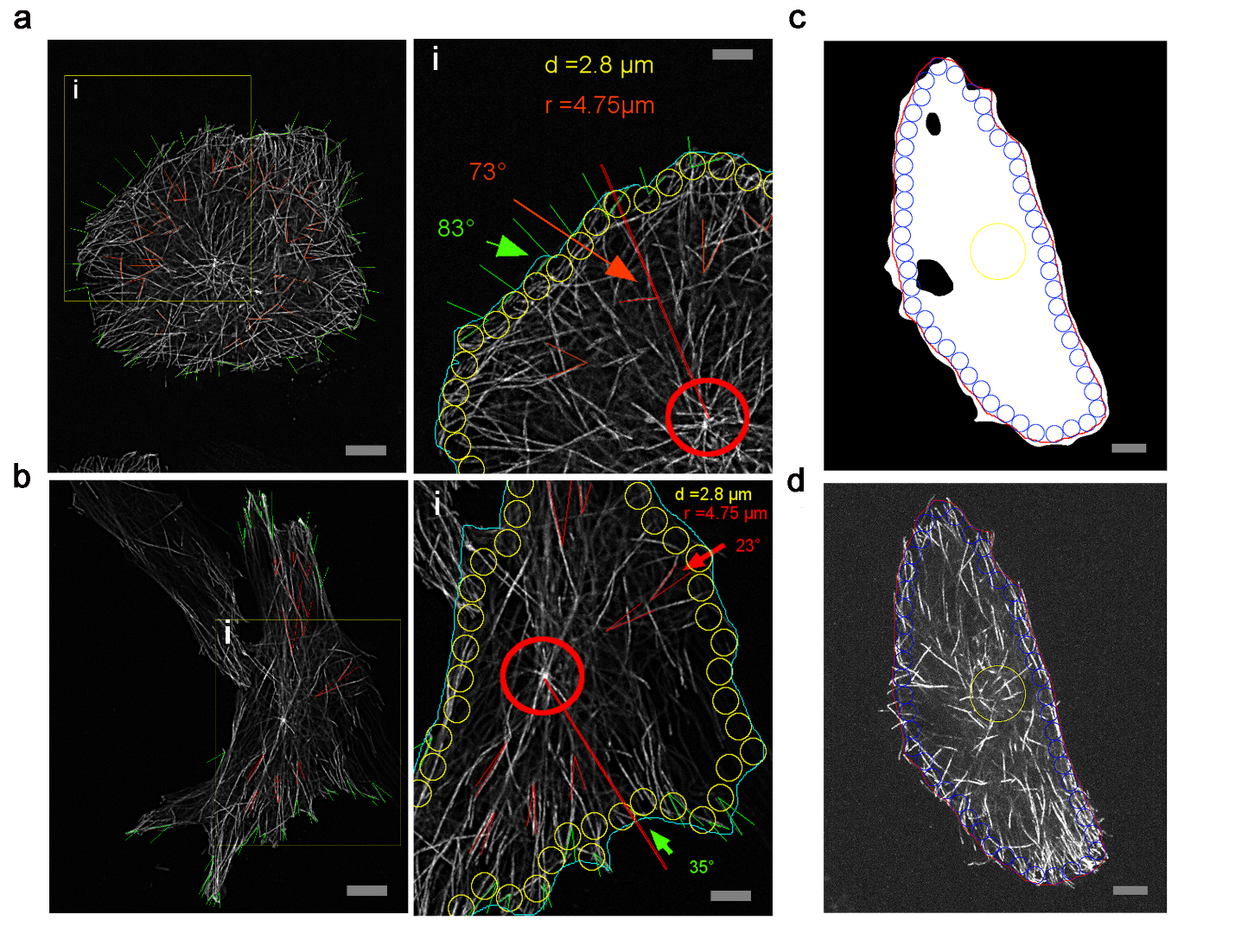


**Figure S9.** The measurement of MTs growth trajectories angles with respect to cell radius and description of zoning in cells. (a) MTs’ growth trajectory angles in cells before EMT. (b) MTs’ growth trajectory angles in cells after EMT. Trajectories were analyzed at the cell edge (at a distance ≤2.8 µm from the cell edge) and in the cell interior (at a distance >4.75 µm from the centrosome and >2.8 µm from the cell edge). The angles of plus-end growing microtubule trajectories were measured relative to the straight line (red) that was derived from the centrosome region to the cell periphery. Measured angles of MTs plus end growth trajectories in the cell interior are shown in red. Measured angles of MTs plus end growth trajectories at the cell margin are shown in green. ­Maximum intensity projections of 20 seconds (10 frames) for EB3-RFP tracks were created using imaging software, Fiji Image J. Scale bar 10µm. (c-d) The cell edge region was determined on maximal intensity projection (MIP) images of cells transfected with EB-3-GFP as the boundary of the area inside which individual growth tracks of MTs frequently overlap. (c) The result of blurring an image by a Gaussian function and converting it to binary. To draw cell boundaries we choose the following procedure:

Open the image of interest in Fiji ImageJ.

Convert to 8-bit image

Apply Gaussian blur with a sigma radius of 8-12 to the image (Proces > Filters > Gaussian Blur).

Process >Binary> Make binary

Select the "Freehand Selection" tool from the toolbar.

Draw a boundary around the cell (red line), where MTs densely distributed and blue circles -the region -“cell-edge” (at a distance ≤2.8 µm from the cell edge), where the length of MTs plus end growth events, angle of growth and the density of MTs distribution was analyzed

Choose Analyze >Tools> ROI Manager >Add

(d) The representation of how drawn regions cell boundary (red line) and “cell-edge” (at a distance ≤2.8 µm from the cell edge) (blue circles) apply on the original 8-bit image without blurring. Scale bar 10µm.
