# Supplementary material for: Morphological and cytoskeleton changes in cells after EMT
Source: Sci Rep. 2023 Dec 13;13:22164. doi: 10.1038/s41598-023-48279-y (PMC10719275; doi:10.1038/s41598-023-48279-y)
Supplement: Supplementary file 10 — Supplementary Figure S10. [file 41598_2023_48279_MOESM10_ESM.docx]

**
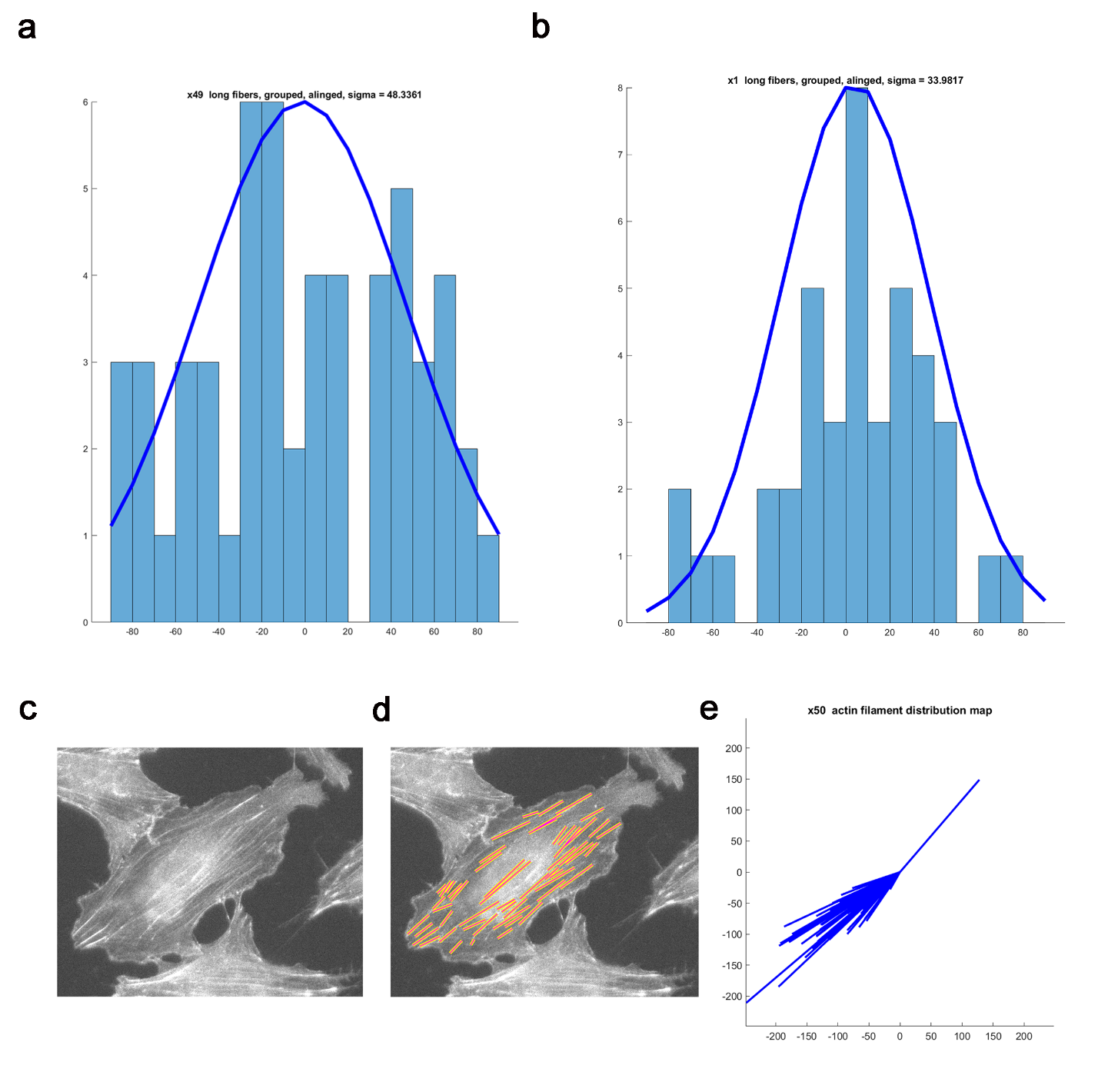
**

**Figure S10.** Graphical representation of an angular distribution of fiber directions. In this histogram, the X-axis represents an angle (in degrees); the Y-axis represents the frequency or count of actin fibers falling within each angle. Since all fibers are bidirectional (i.e. a fiber facing at 0^0^ is simultaneously also facing at 180^0^), all angles are shown on a -90^0^ to +90^0^ scale. The base direction from which angles are measured can be selected arbitrarily, so for each cell, this direction (0^0^ angle) is selected algorithmically to better center the distribution around it. The calculated sigma represents the width of the normal fit of the distribution. A larger sigma (σ) value indicates a wider distribution of fibers, suggesting less co-oriented fiber organization (a). Conversely, a smaller sigma value indicates a narrower distribution and implies a more homogeneous or uniform distribution of fiber directions, meaning a more co-oriented fiber distribution (b). (c) Widefield image of the analyzed cell. (d) Image of cell with actin filaments pointed out in red. (e) The graphical illustration of actin filament direction relative to one another. The X/Y axis shows the fiber scale (in pixels). This graph was generated using the Matlab script described in Material and Methods.
