# Supplementary material for: Morphological and cytoskeleton changes in cells after EMT
Source: Sci Rep. 2023 Dec 13;13:22164. doi: 10.1038/s41598-023-48279-y (PMC10719275; doi:10.1038/s41598-023-48279-y)
Supplement: Supplementary file 11 — Supplementary Figure S11. [file 41598_2023_48279_MOESM11_ESM.docx]

**
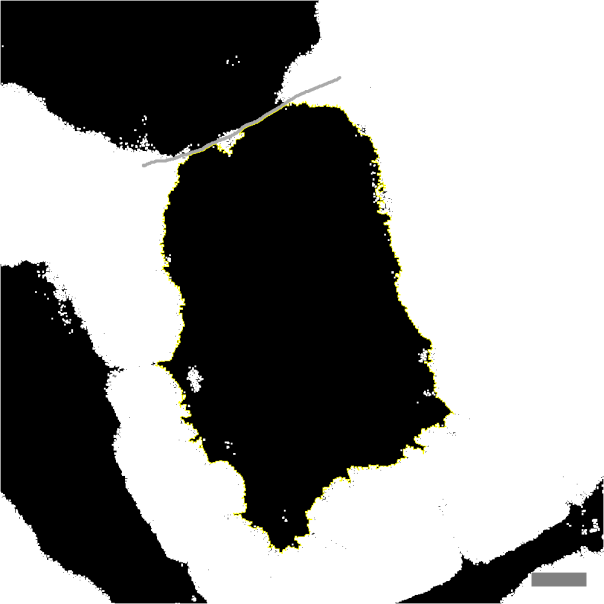
**

**Figure S11.** Area measurements of cells using Fiji ImageJ software. Scale bar 10µm.

To measure cell area in Fiji ImageJ we choose the following procedure:

Open the image of interest in Fiji ImageJ.

Convert to 8-bit image

Process >Binary> Make binary

Process >Binary>Close

Chose “Paintbrush tool “and draw a line to separate adjacent cells

Select “wand (tracing) tool”

Click the cell we want to analyze

Choose Analyze >Tools> ROI Manager >Add>Measure
