# Supplementary material for: Morphological and cytoskeleton changes in cells after EMT
Source: Sci Rep. 2023 Dec 13;13:22164. doi: 10.1038/s41598-023-48279-y (PMC10719275; doi:10.1038/s41598-023-48279-y)
Supplement: Supplementary file 12 — Supplementary Figure S12. [file 41598_2023_48279_MOESM12_ESM.docx]

**
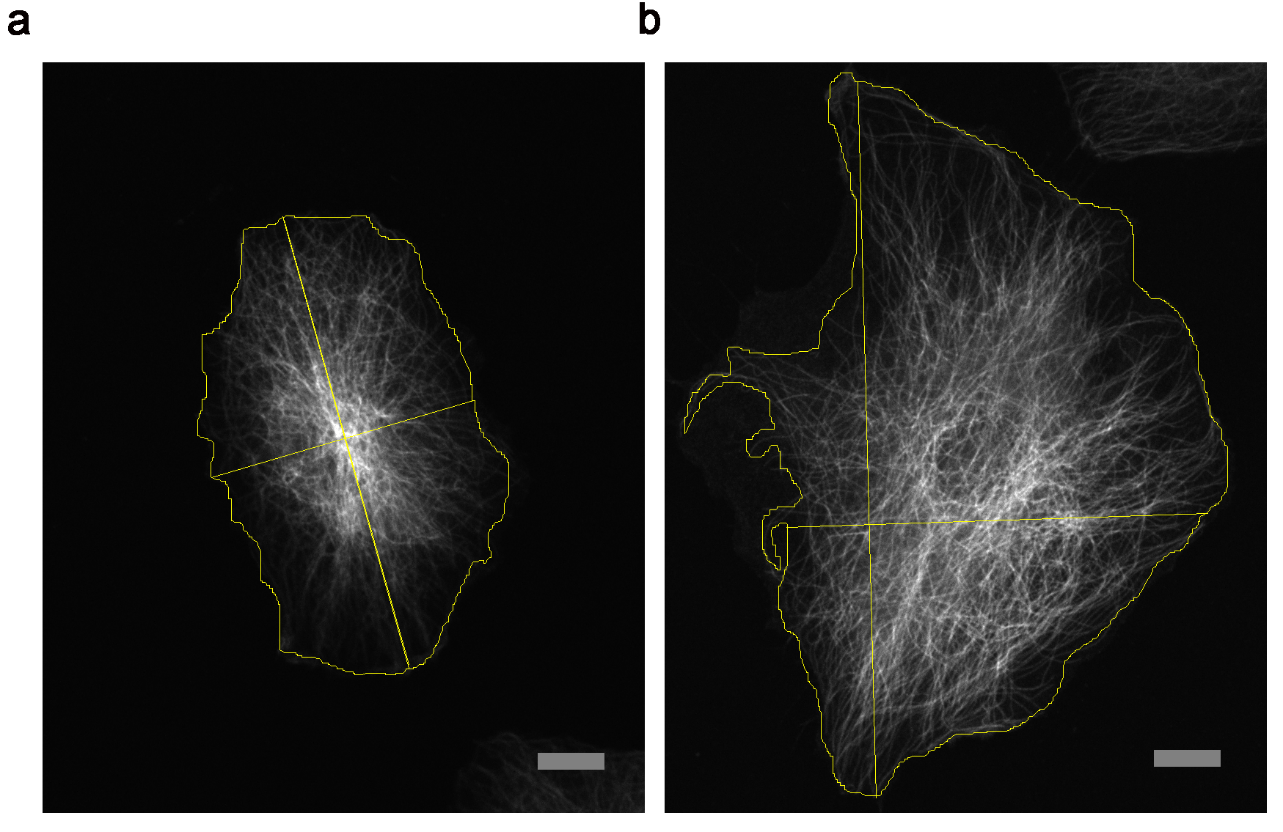
**

**Figure S12.** The aspect ratio measurement. (a) Cell that resembles oval shape. The major axis is the longest axis inside the cell. The minor axis is perpendicular to the longest axis in the middle of the cell. (b) Cell shape is different from an oval. The major axis is the longest axis inside the cell. The minor axis is randomly chosen perpendicular to the longest axis and it is maximally located close to the relative cell center. Scale bar 10µm.
