# Supplementary material for: Morphological and cytoskeleton changes in cells after EMT
Source: Sci Rep. 2023 Dec 13;13:22164. doi: 10.1038/s41598-023-48279-y (PMC10719275; doi:10.1038/s41598-023-48279-y)
Supplement: Supplementary file 13 — Supplementary Figure S13. [file 41598_2023_48279_MOESM13_ESM.docx]

–
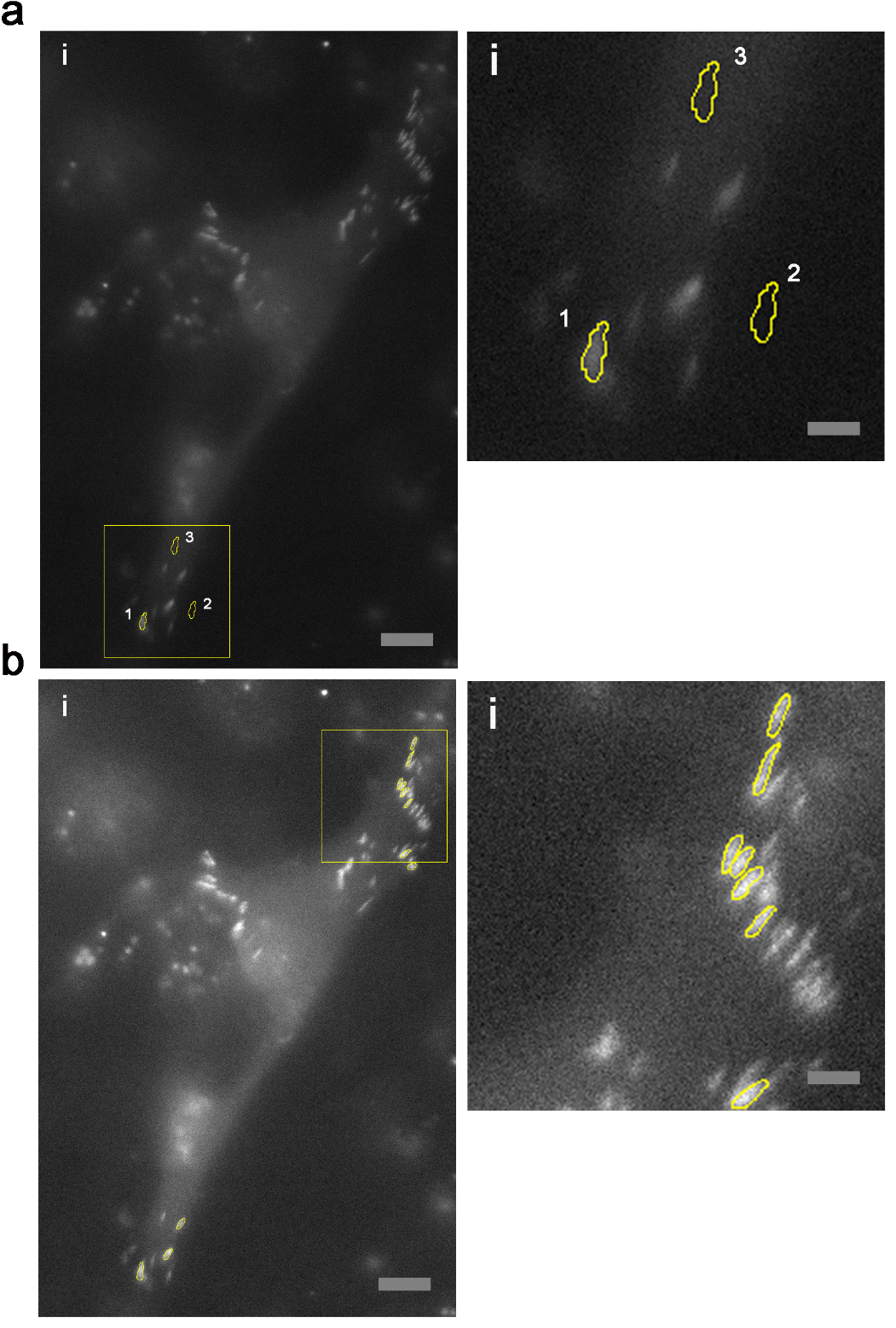


**Figure S13.** Measurement of the characteristics of FAs. Scale bar 10µm.

To measure the area, integrated brightness and protein enrichment coefficient of FAs in Fiji [ImageJ] the following procedure was chosen:

Open the image of interest in ImageJ.

Analyze > Set scale > Distance in pixels=0; Known distance=0; Pixel aspect ratio=1; Unit of length - pixels; Select global.

Analyze > Set measurements > Select Area> Select Integrated brightness

Select the "Freehand Selection" tool from the toolbar.

Draw a boundary around the FA where borders clearly can be seen (Figure S12 a (i1)).

Choose Analyze >Tools> ROI Manager >Add

Choose regions of the same size in cytoplasm (Figure S12 a (i3)) and outside of the cell (Figure S12 a (i2)). > ROI Manager >Add>Measure

Integrated brightness of FA = A-C, where A-(measured area of FA (pixels) (Figure S12 a (i1)) and C-measured area in cytoplasm with the same area as for the FA (pixels) (Figure S12 a (i3)).

Protein enrichment coefficient = (A-C)/(C-B), where A is integrated brightness of FA (pixels) (Figure S12 a (i1)), C is integrated brightness of the cytoplasm with the same area as for the FA (pixels) (Figure S12 a (i3)) and B is integrated brightness of background with the same area as for the FA (pixels) (Figure S12 a (i2)).
