# Supplementary material for: Morphological and cytoskeleton changes in cells after EMT
Source: Sci Rep. 2023 Dec 13;13:22164. doi: 10.1038/s41598-023-48279-y (PMC10719275; doi:10.1038/s41598-023-48279-y)
Supplement: Supplementary file 29 — Supplementary Table S1. [file 41598_2023_48279_MOESM29_ESM.docx]

**Table S1.** Alterations in size and aspect ratio of cells after EMT

| **Cells** | **before/**  **after EMT** | **Area (µm^2^), mean ± SD** | **Statistics**  **(Mann-Whitney U test)** | **Variability (S.D./mean, %)** | **Aspect/ratio,**  **mean ± SD** | **Statistics**  **(Mann-Whitney U test)** | **Variability (S.D./mean, %)** | **N=cells** |
| --- | --- | --- | --- | --- | --- | --- | --- | --- |
| MCF-7 | Before | 1582 ± 877 | p=0.03 | 55 | 1.5 ± 0.6 | p <0.0001 | 40 | 105 |
|  | After | 2309 ± 1808 |  | 78 | 2.3 ± 1.8 |  | 78 | 107 |
| A-549 | Before | 2460 ± 1755 | p=0.01 | 71 | 2.0 ± 0.7 | p <0.0001 | 35 | 108 |
|  | After | 3082 ± 2448 |  | 79 | 3.3 ± 1.6 |  | 48 | 100 |
| HaCaT | Before | 1942 ± 1651 | p<0.0001 | 85 | 1.8 ± 0.7 | n.s | 39 | 103 |
|  | After | 3743 ± 2972 |  | 79 | 1.7 ± 0.7 |  | 41 | 100 |
