# Supplementary material for: Morphological and cytoskeleton changes in cells after EMT
Source: Sci Rep. 2023 Dec 13;13:22164. doi: 10.1038/s41598-023-48279-y (PMC10719275; doi:10.1038/s41598-023-48279-y)
Supplement: Supplementary file 30 — Supplementary Table S2. [file 41598_2023_48279_MOESM30_ESM.docx]

**Table S2.** The differences in MTs’ plus end growth speed in different cancer cells before and after EMT

| **Cells** | **before/**  **after EMT** | **MTs’ plus end growth speed (µm/min), mean ± SD** | **Statistics**  **(t-test)** | **MTs’ plus end growth track (µm)**  **mean ± SD** | **Statistics**  **(t-test)** | **N=cells** | **N=comets** |
| --- | --- | --- | --- | --- | --- | --- | --- |
| MCF-7 | Before | 20.25 ± 10.68 | p <0.0001 | 5.9 ± 5.2 | p <0.0001 | 21 | 311 |
|  | After | 23.36 ± 10.56 |  | 6.8 ± 6.0 |  | 24 | 291 |
| A-549 | Before | 29.01 ± 10.13 | p <0.0001 | 6.5 ± 6.9 | p <0.0001 | 10 | 200 |
|  | After | 32.82 ± 9.83 |  | 9.7 ± 10.1 |  | 10 | 200 |
| HaCaT | Before | 22.30 ± 20.66 | p<0.0001 | 4.7 ± 4.9 | p <0.0001 | 11 | 415 |
|  | After | 27.02 ± 20.09 |  | 6.8 ± 7.2 |  | 11 | 415 |
