# Supplementary material for: Morphological and cytoskeleton changes in cells after EMT
Source: Sci Rep. 2023 Dec 13;13:22164. doi: 10.1038/s41598-023-48279-y (PMC10719275; doi:10.1038/s41598-023-48279-y)
Supplement: Supplementary file 31 — Supplementary Table S3. [file 41598_2023_48279_MOESM31_ESM.docx]

**Table S3.** The differences in MTs’ characteristics in different cells before and after EMT.

| **Characteristics** |  | **MCF-7** | | **Statistics**  **(Mann-Whitney U test)** | **A-549** | | **Statistics**  **(Mann-Whitney U test)** | **HaCaT** | | **Statistics**  **(Mann-Whitney U test)** |
| --- | --- | --- | --- | --- | --- | --- | --- | --- | --- | --- |
|  |  | **Before EMT** | **After EMT** |  | **Before EMT** | **After EMT** |  | **Before EMT** | **After EMT** |  |
| Length of plus end growing MTs ( µm),  mean ± SD | cell interior | 3.43 ± 1.46 (N=11 cells, 160 MTs) | 4.85 ± 1.91 (N=16 cells,  158 MTs) | p <0.0001 | 5.74 ± 2.09  (N=12 cells, 161 MTs) | 6.34 ± 2.43  (N=12 cells, 104 MTs) | p =0.05 | 4.27 ± 1.76  (N=10 cells,  80 MTs) | 6.73 ± 3.02  (N=10 cells,  62 MTs) | p <0.0001 |
|  | cell margin | 2.98 ± 1.24 (N=11 cells, 127 MTs) | 5.09 ± 2.07 (N=16 cells,  174 MTs) | p <0.0001 | 5.77 ± 1.97  (N=7 cells, 57 MTs) | 7.44 ± 2. 05  (N=6 cells, 44 MTs) | p <0.0002 | 3.64 ± 1.76  (N=10 cells, 71  MTs) | 6.50 ± 2.81  (N=10 cells, 69 MTs) | p <0.0001 |
| Density of comets per 1 µm² | cell interior | 0.46  (N=11 cells, 246 MTs) | 0.47  (N=17 cells, 257 MTs) | ns | 0.51  (N=22 cells, 301 MTs) | 0.61  (N=18 cells, 252 MTs) | ns | 0.81  (N=18 cells, 323 MTs) | 0.92  (N=17 cells, 348 MTs) | ns |
|  | cell margin | 0.45  (N=11 cells, 423 MTs) | 0.43  (N=17 cells, 445 MTs) | ns | 0.56  (N=22 cells, 507 MTs | 0.61  (N=18 cells, 414 MTs) | ns | 0.89  (N=18 cells, 1031 MTs) | 0.81  (N=17 cells, 1035 MTs) | p =0.02 |
| Angle (°),  mean ± SD | cell interior | 57 ± 29  (26 cells,  144 MTs) | 41 ± 26  (24 cells,  162 MTs) | p<0.0001 | 53 ± 33  (10 cells,  48 MTs) | 37 ± 33  (9 cells,  46 MTs) | p <0.01 | 56 ± 30  (16 cells, 89 MTs) | 49 ± 28  (17 cells, 87 MTs) | ns |
|  | cell margin | 59 ± 28  (N=26 cells, 185 MTs) | 53 ± 30  (N=24 cells, 278 MTs) | p <0.01 | 57 ± 34  (N=10 cells, 52 MTs) | 28 ± 26  (N=9 cells, 47 MTs) | p<0.0001 | 54 ± 28  (N=16 cells, 157 MTs) | 42 ± 27  (N=17 cells, 138 MTs) | p <0.0001 |
