# Supplementary material for: Morphological and cytoskeleton changes in cells after EMT
Source: Sci Rep. 2023 Dec 13;13:22164. doi: 10.1038/s41598-023-48279-y (PMC10719275; doi:10.1038/s41598-023-48279-y)
Supplement: Supplementary file 32 — Supplementary Table S4. [file 41598_2023_48279_MOESM32_ESM.docx]

**Table S4.** The differences in FAs’ characteristics in different cancer cells before and after EMT

| **Characteristics** | **MCF-7** | | **Statistics**  **(Mann-Whitney U test)** | **A-549** | | **Statistics**  **(Mann-Whitney U test)** | **HaCaT** | | **Statistics**  **(Mann-Whitney U test)** |
| --- | --- | --- | --- | --- | --- | --- | --- | --- | --- |
|  | **Before EMT** | **After EMT** |  | **Before EMT** | **After EMT** |  | **Before EMT** | **After EMT** |  |
| Integrated brightness (a.u), median | 14298 | 20859 | p=0.002 | 16967 | 18745 | ns | 13736 | 5546 | p< 0.0001 |
| Protein enrichment coefficient,  median | 7.47 | 7.07 | ns | 5.60 | 5.62 | ns | 3.51 | 3.99 | ns |
| Area ( µm²), median | 1.13 | 0.94 | p=0.001 | 1.97 | 1.41 | p< 0.0001 | 1.03 | 0.80 | p< 0.0001 |
| Lifetime (minutes), median | 14 | 18 | p=0.002 | 20 | 34 | p< 0.0001 | 27 | 18 | p< 0.0001 |
| Number of FAs per cell,  median | 25 | 20 | ns | 54 | 46 | ns | 30 | 50 | p< 0.0001 |
| N=cells/FAs | 10/90 | 7/66 |  | 17/165 | 16/139 |  | 20/195 | 14/135 |  |
