# Supplementary material for: Morphological and cytoskeleton changes in cells after EMT
Source: Sci Rep. 2023 Dec 13;13:22164. doi: 10.1038/s41598-023-48279-y (PMC10719275; doi:10.1038/s41598-023-48279-y)
Supplement: Supplementary file 33 — Supplementary Table S5. [file 41598_2023_48279_MOESM33_ESM.docx]

**Table S5.** Primer details

| **№** | **Name** | **Position** | **5'-3' Sequence** | **Annealing Temperature (°C )** | **Length (bp)** |
| --- | --- | --- | --- | --- | --- |
| 1 | SNAIL F | Forward | CGCTCTTTCCTCGTCAGGA | 57 | 19 |
| 2 | SNAIL R | Reverse | GTCCCAGATGAGCATTGGC | 57 | 19 |
| 3 | SLUG F | Forward | CCCTCACTGCAACAGAGCAT | 59 | 20 |
| 4 | SLUG R | Reverse | TACACAGCAGCCAGATTCCTC | 59 | 21 |
| 5 | E-cadherin F | Forward | TGGAACAGGGACACTTCTGC | 62 | 20 |
| 6 | E-cadherin R | Reverse | CCCGTGTGTTAGTTCTGCTGT | 62 | 21 |
| 7 | N-cadherin F | Forward | TGTGACCGATAAGGATCAACC | 61 | 21 |
| 8 | N-cadherin R | Reverse | ACTAACCCGTCGTTGCTGTT | 61 | 20 |
| 9 | Vimentin F | Forward | ACCTGTGAAGTGGATGCCCT | 58 | 20 |
| 10 | Vimentin R | Reverse | ACGAAGGTGACGAGCCATTT | 58 | 20 |
| 11 | TWIST1 F | Forward | GGAGTCCGCAGTCTTACGA | 58 | 19 |
| 12 | TWIST1 R | Reverse | GTCTGAATCTTGCTCAGCTTGT | 58 | 22 |
| 13 | Beta III Tubulin F | Forward | TCAGCGTCTACTACAACGAGGC | 59 | 22 |
| 14 | Beta III Tubulin R | Reverse | GCCTGAAGAGATGTCCAAAGGC | 59 | 22 |
| 15 | Beta I Tubulin F | Forward | TAGCTTTTCTCCTGACTGGC | 58 | 20 |
| 16 | Beta I Tubulin R | Reverse | AATCGGCAGAATGAGTTGAC | 58 | 20 |
